# Supplementary material for: EMPOWERED trial: protocol for a randomised control trial of digitally supported, highly personalised and measurement-based care to improve functional outcomes in young people with mood disorders
Source: BMJ Open. 2023 Oct 11;13(10):e072082. doi: 10.1136/bmjopen-2023-072082 (PMC10583041; doi:10.1136/bmjopen-2023-072082)
Supplement: Supplementary data [file bmjopen-2023-072082supp001.pdf]

THE UNIVERSITY OF SYDNEY
Brain and Mind Centre

Participant Information Sheet/Consent Form

Interventional Study - *Adult providing own consent*

Mind Plasticity – Brain and Mind Centre

**Title**	A large-scale clinical effectiveness (health services) trial to determine whether personalised health care packages, combined with digitally-supported measurement-based care, improve functional outcomes in young people with mood disorders.
**Short Title**	EMPOWERED Trial.
**Protocol Number**	BMC-YMH-003-2018
**Project Sponsor**	The University of Sydney
**Coordinating Principal Investigator/ Principal Investigator**	Professor Ian B. Hickie
**Location**	Brain and Mind Centre, University of Sydney<br>Mind Plasticity, Surry Hills

Part 1 What does my participation involve?

1 Introduction

You are invited to take part in this research project which is investigating whether more personalised health care packages, linked with continuous digital feedback, deliver better functional improvements at 12 months (and follow-up for a further 12 months after cessation of active care) than digitally-supported assessment linked to standard care packages.

This Participant Information Sheet (PIS) tells you about the research project. It explains the tests and treatments involved. Knowing what is involved will help you decide if you want to take part in the research.

Please read this information carefully. Ask questions about anything that you don't understand or want to know more about. Before deciding whether or not to take part, you might want to talk about it with a relative, friend, or your local doctor.

Participation in this research is voluntary. If you don't wish to take part, you don't have to. You will receive the best possible care whether or not you take part.

Hickie IB, et al. BMJ Open 2023; 13:e072082. doi: 10.1136/bmjopen-2023-072082

If you decide you want to take part in the research project, you will be asked to sign the consent section. By signing it you are telling us that you:

• Understand what you have read
• Consent to take part in the research project
• Consent to have the tests and treatments that are described
• Consent to the use of your personal and health information as described.

You will be given a copy of this Participant Information and Consent Form to keep.

2 What is the purpose of this research?

This study aims to assess in 1500 young people with mood syndromes, whether more personalised health care packages, linked with continuous digital feedback (i.e. highly personalised and measurement based care (HP&MBC), deliver better functional improvements at 12 months (and follow-up for a further 12 months after cessation of active care), than digitally-supported assessment linked to standard care packages.

Although the standard care packages are an improved offering (through greater standardisation of assessment and feedback of those assessments), we hypothesise that the HP&MBC treatment packages are superior, by implementing continuous monitoring and care coordination through the use of digital technologies, and providing extensive feedback to the clinical service, the treating clinician, and the young person and their family or carer. The continuous feedback will detect unmet care, increase the likelihood of identifying young persons that do not respond to treatment, and facilitate the process to optimise care and increase the engagement of young people in their own care.

This research has been initiated by the investigator, Professor Ian B. Hickie, Co-Director and Consultant Psychiatrist, Brain and Mind Centre, The University of Sydney. This research is being conducted by the Brain and Mind Centre and Mind Plasticity. The study has been funded by NHMRC 2020 Clinical Trials and Cohort Studies (Application ID: 2001568).

3 What does participation in this research involve?

If you consent to participate, you will be taking part in a randomised controlled research project. Sometimes we do not know which treatment is best for treating a condition. To find out we need to compare different treatments. We put people into groups and give each group a different treatment. The results are compared to see if one is better. To try to make sure the groups are the same, each participant is put into a group by chance (at random).

You have been invited to take part in this trial as your clinician indicated that you may be eligible and interested in taking part. This clinical trial comprises 12-months of an active treatment care package and a 12-month follow-up phase, meaning the duration of the trial is expected to be 24 months from your baseline visit. If you decide to take part, an appointment will be scheduled for an enrolment visit at the Brain and Mind Centre (BMC).

Enrolment Visit *(time commitment: 60 minutes)*

During your enrolment visit, a staff member will confirm that you have read and understood this PIS, the study will also be verbally explained to you, and you will be given the opportunity to ask any questions you may have. If you consent to participating you will be asked to sign a written consent form.

Once your consent is provided, you will be asked some relevant questions to confirm that you meet the other inclusion and exclusion criteria for the study. If you are eligible to take part you will be invited to attend a baseline visit.

Once enrolled in the study, you will be randomly assigned to receive one of two treatment packages for a 12 month duration.

You will have approximately equal chance of being assigned to one of the following two care packages:

1. The *Highly Personalised and Measurement Based Care (HP&MBC)* Package: This includes:
   - Initial e-health assessment with feedback provided to yourself, your treating clinician, and the clinical service.
   - Continuous monitoring via e-health assessment and monthly feedback over the 12-month treatment duration to yourself, your treating clinician, and the clinical service.
   - Personalised referrals over the 12-month treatment duration, to specific treatment programs that may be beneficial to you,based on the outcome of continuous assessment data.

2. *Standardised care package:* This includes:
   - Initial e-health assessment with feedback provided to yourself, your treating clinician, and the clinical service.
   - Provision of standard multidisciplinary care options and ongoing access to other relevant psychological and pharmacological options.
   - Additional e-health assessments at 3, 6 and 12-months.

Baseline Visit *(time commitment: 60-90 minutes)*

During your baseline visit, you will be asked questions about your day-to-day activities, mood, and behaviour.

You will also be asked to complete a series of online self-report assessments that will include further questions about your mental health symptoms, physical activity & physical health, sleep, and the quality of your relationships and social supports. These self-report questions will be accessed online and completed via iPad.

After the baseline assessment, you will be required to visit your local pathology centre for a blood test for the assessment of metabolic, inflammatory, and standard blood markers. The results will be sent to the study doctor.

Follow-up assessments (3, 6, 12, 24 months) (*time commitment: 60-90 minutes)*

You will be invited back for follow-up research assessments 3, 6, 12 and 24 months after the commencement of your treatment. Interview and online questionnaire assessments will again be conducted, including questions about your day-to-day activities, mood, and behaviour.

At the **6 & 12-month** time points you will also be required to visit your local pathology centre for a blood test which will again be sent to the study doctor.

Hickie IB, *et al. BMJ Open* 2023; 13:e072082. doi: 10.1136/bmjopen-2023-072082

Additional Costs

Throughout the research project specific treatment programs may be recommended to you based on the outcome of continuous assessment data. There may be additional costs associated with these treatment options. Any additional costs will be discussed with you in advance, and you will have the option to decline.

If you have a local doctor, we recommend that you inform them of your participation in this research project. In addition, the researchers would like to have access to your medical record to obtain information relevant to this study.

You will be reimbursed for your time in this study. Reimbursement will be $50 and this will be provided to you in the form of a Coles/Myer voucher.

4 What do I have to do?

To participate in this study you must meet some criteria, including:

- Aged 15-25 years old, seeking help for psychological distress
- Classified as suitable for the intervention based on the enrolment assessment
- Written informed consent

You will *not* be able to participate in the study if you meet certain criteria, including:

- Acute suicidal or aggressive behaviour requiring alternative care
- Depressive syndrome secondary to a primary medical condition
- Intellectual disability

5 Other relevant information about the research project

This study will involve 1500 participants, and will primarily be conducted at The Brain and Mind Centre, University of Sydney, 94-100 Mallet Street, Camperdown and Mind Plasticity, Suite 517, Level 5, 50 Holt St, Surry Hills NSW 2010.

6 Do I have to take part in this research project?

Participation in any research project is voluntary. If you do not wish to take part, you do not have to. If you decide to take part and later change your mind, you are free to withdraw from the project at any stage; you don't have to give a reason. If you do want to take part now, but change our mind later, you can pull out of the study at any time.

If you do decide to take part, you will be given this Participant Information and Consent Form to sign and you will be given a copy to keep.

Hickie IB, *et al. BMJ Open* 2023; 13:e072082. doi: 10.1136/bmjopen-2023-072082

Your decision whether to take part or not to take part, or to take part and then withdraw, will not affect your relationship with those treating you or your relationship with the Mind Plasticity and/or Brain and Mind Centre, The University of Sydney.

7 What are the alternatives to participation?

You do not have to take part in this research project to receive treatment. Participation in this research is not your only option. Other options are available; these include engaging in / continuing with the standard treatment options offered. The study doctor will discuss these options with you before you decide whether or not to take part in this research project. You can also discuss the options with your local doctor and/or treating clinician.

8 What are the possible benefits of taking part?

We cannot guarantee or promise that you will receive any direct benefits from being in this study. However, the aim of this study is to further the knowledge and treatment of mood disorders in young people.

9 What are the possible risks and disadvantages of taking part?

We anticipate that the possible risks associated with this study are no more than low risk. You may feel anxious during some of the assessment procedures. If you become upset or distressed as a result of your participation in the research, the study doctor will be able to arrange for counselling or other appropriate support. Any counselling or support will be provided by a qualified staff who are not members of the research project team.

Having a blood sample taken may cause some discomfort, bruising, minor infection or bleeding. If this happens, it can be easily treated by your local doctor.

The study doctor will review the results of your blood test. In the event that the results of your blood test require further investigation, you will be referred to your local doctor.

10 What will happen to my test samples?

You will be asked to provide blood samples at baseline, 6 and 12 months post baseline. Blood samples will be collected in a fasting state by a trained phlebotomist at your local pathology centre. Standard infection control procedures will be followed to avoid harm to participants. Blood samples will be used to identify the following:

- Metabolic blood measures
- Inflammatory markers
- Standard clinical blood measures

All blood test results will be labelled with your study ID code only, and no identifying information.

Your data can only be obtained and used by researchers who have their study approved by a Human Research Ethics Committee. Any scientists who wish to use your data must also agree to protect your privacy and store data securely.

All blood test results will be reviewed by the study doctor. If there are any abnormalities identified in the blood results, you will be notified and asked to see your local treating GP for

Hickie IB, et al. BMJ Open 2023; 13:e072082. doi: 10.1136/bmjopen-2023-072082

review. Blood samples will not be stored as part of this study and therefore samples will not be retained for future use.

11 What if new information arises during this research project?

Sometimes during the course of a research project, new information becomes available about the treatment that is being studied. If this happens, your study doctor will tell you about it and discuss with you whether you want to continue in the research project. If you decide to withdraw, your study doctor will make arrangements for your regular health care to continue. If you decide to continue in the research project you will be asked to sign an updated consent form.

Also, on receiving new information, your study doctor might consider it to be in your best interests to withdraw you from the research project. If this happens, he/ she will explain the reasons and arrange for your regular health care to continue.

12 What if I withdraw from this research project?

You can withdraw from the study at any time by contacting research staff. Your decision whether to participate will not affect your current or future relationship with the researchers or anyone else at The University of Sydney nor your current or future involvement with the mental health service.

If you decide to withdraw from the project, please notify a member of the research team before you withdraw. This notice will allow that person or the research supervisor to discuss any health risks or special requirements linked to withdrawing.

You will be asked about the reason(s) for your discontinuation and about the presence of any adverse events. You will be invited to attend the Brain and Mind Centre for a final close-out visit to complete all assessments normally completed at the final study visit (i.e., 12-months post baseline).

Further, relevant information about your health status as judged by the investigator, which comes up after the screening visit may justify a subsequent exclusion from the study. Relevant information could be information concerning inclusion or exclusion criteria or information that implies that the treatment schedule is not suitable.

If you do withdraw your consent during the research project, relevant study staff will not collect additional personal information from you, although personal information already collected will be retained to ensure that the results of the research project can be measured properly and to comply with law. You should be aware that data collected up to the time you withdraw will form part of the research project results. If you do not want them to do this, you must tell them before you join the research project.

13 Could this research project be stopped unexpectedly?

In the unlikely event that the local regulatory/health authorities suspend the trial, you would still be able to access your treatment as usual. It won’t impact your relationship with the University of Sydney or the service from which you are accessing care.

Part 2 How is the research project being conducted?

14 What will happen to information about me?

By signing the consent form you consent to the study doctor and relevant research staff collecting and using personal information about you for the research project. Any information obtained in connection with this research project that can identify you will remain confidential. Your information will only be used for the purpose of this research project and it will only be disclosed with your permission, except as required by law.

Information about you may be obtained from your health records held at this and other health services for the purpose of this research. By signing the consent form you agree to the study team accessing health records if they are relevant to your participation in this research project.

Your health records and any information obtained during the research project are subject to inspection (for the purpose of verifying the procedures and the data) by the relevant authorities and authorised representatives of the Sponsor, The University of Sydney, or as required by law. By signing the Consent Form, you authorise release of, or access to, this confidential information to the relevant study personnel and regulatory authorities as noted above.

It is anticipated that the results of this research project will be published and/or presented in a variety of forums. In any publication and/or presentation, information will be provided in such a way that you cannot be identified, except with your permission.

Information about your participation in this research project may be recorded in your health records.

In accordance with relevant Australian and NSW privacy and other relevant laws, you have the right to request access to your information collected and stored by the research team. You also have the right to request that any information with which you disagree be corrected. Please contact the study team member named at the end of this document if you would like to access your information.

Any information obtained for the purpose of this research project and for the future research described in Section 14 that can identify you will be treated as confidential and securely stored. It will be disclosed only with your permission, or as required by law.

All study records will be stored for a minimum of 20 years post study completion and then securely destroyed.

All data collected for the purposes of the study will be linked to unique study ID codes and will not contain identifying information. Data will be stored separately from any identifying information (e.g., signed consent forms). One senior research staff member at each site will have an electronic password protected file linking participant names and identification codes (i.e., data will be re-identifiable). Individuals will not be named in any reports or publications resulting from the study, and no document containing identifying information will leave the study site. Any publications based on the study will include only pooled results from participants.

Data collection will be conducted only by authorised members of study staff, to whom this duty has been allocated and who are named on the Human Ethics application and Governance approvals for the trial. Only sufficiently trained and supervised research staff will be delegated to enter and analyse data. We will be using a RedCap database to enter the research data. Data for which hardcopies are generated will be stored in both original hard copy and electronic form. This data will be kept under 1) lock and key at trial site or 2) electronic file that is password

protected and accessible only by research staff responsible for data entry or monitoring. Electronic data generated by participant outcomes will be electronically stored via the *Innowell Online Platform*. The information stored via this online platform will be de-identified and subject to privacy policies and the Research Code of Conduct. All data will be analysed by study staff at the Brain and Mind Centre, The University of Sydney. There will be no sharing or pooling of data with other collaborators.

With consent, we may use data collected in this study for future research purposes.

15 Complaints and compensation

If you suffer any injuries or complications as a result of this research project, you should contact the study team as soon as possible and you will be assisted with arranging appropriate medical treatment. If you are eligible for Medicare, you can receive any medical treatment required to treat the injury or complication, free of charge, as a public patient in any Australian public hospital.

In addition, you may have a right to take legal action to obtain compensation for any injuries or complications resulting from the study. Compensation may be available if your injury or complication is sufficiently serious and is caused by unsafe equipment, or by the negligence of one of the parties involved in the study (for example, the researcher, the clinic, or the treating clinician). You do not give up any legal rights to compensation by participating in this study.

16 Who is organising and funding the research?

This research project is being conducted by Professor Ian Hickie at The Brain and Mind Centre, Sydney University.

This trial is an investigator-initiated trial funded by the NHMRC – 2020 Clinical Trials and Cohort Studies, Application ID: 2001568.

You will not benefit financially from your involvement in this research project.

No member of the research team will receive a personal financial benefit from your involvement in this research project (other than their ordinary wages).

17 Who has reviewed the research project?

All research in Australia involving humans is reviewed by an independent group of people called a Human Research Ethics Committee (HREC). The ethical aspects of this research project have been approved by the HREC of the Sydney Local Health District (RPAH Zone). This research project has been designed to make sure the researchers interpret the results in a fair and appropriate way and avoid study researchers or participants jumping to conclusions.

This project will be carried out according to the *National Statement on Ethical Conduct in Human Research (2007)*. This statement has been developed to protect the interests of people who agree to participate in human research studies.

18 Further information and who to contact

The person you may need to contact will depend on the nature of your query.

Hickie IB, *et al. BMJ Open* 2023; 13:e072082. doi: 10.1136/bmjopen-2023-072082

Clinical contact person

Name	Professor Ian Hickie
Position	Principal Investigator
Telephone	(02) 93510810
Email	Ian.hickie@sydney.edu.au

For matters relating to research at the site at which you are participating, the details of the local site complaints person are:

Complaints contact person/s

Name	Ms Alissa Nichles, Ms Natalia Zmicerevska
Position	Senior Clinical research Officers
Telephone	(02) 9114 4100
Email	Alissa.nichles@sydney.edu.au, Natalia.zmicerevska@sydney.edu.au

If you have any complaints about any aspect of the project, the way it is being conducted or any questions about being a research participant in general, then you may contact:

This study has been approved by the Ethics Review Committee (RPAH Zone) of the Sydney Local Health District. Any person with concerns or complaints about the conduct of this study should contact the Executive Officer on 02 9515 6766 and quote Protocol number X22-0042.

Hickie IB, *et al. BMJ Open* 2023; 13:e072082. doi: 10.1136/bmjopen-2023-072082

Consent Form - *Adult providing own consent*

**Title**	A large-scale clinical effectiveness (health services) trial to determine whether personal-ised health care packages, combined with digitally-supported measurement-based care, improve functional outcomes in young people with mood disorders.
**Short Title**	EMPOWERED Trial.
**Protocol Number**	BMC-YMH-003-2018
**Project Sponsor**	The University of Sydney
**Coordinating Principal Investigator/ Principal Investigator**	Professor Ian B. Hickie
**Location**	Brain and Mind Centre, University of Sydney<br>Mind Plasticity, Surry Hills

Declaration by Participant

I have read the Participant Information Sheet or someone has read it to me in a language that I understand.

I understand the purposes, procedures and risks of the research described in the project.

I give permission for my doctors, other health professionals, hospitals or laboratories outside this hospital to release information to Brain and Mind Centre, University of Sydney concerning my disease and treatment for the purposes of this project. I understand that such information will remain confidential.

I understand that my participation in this study will allow the researchers and others, as described in the Information for Participants, to have access to my medical record, and I agree to this.

I have had an opportunity to ask questions and I am satisfied with the answers I have received.

I freely agree to participate in this research project as described and understand that I am free to withdraw at any time during the study without affecting my future health care.

I understand that I will be given a signed copy of this document to keep.

I understand that, if I decide to discontinue the study treatment, I may be asked to attend follow-up visits to allow collection of information regarding my health status. Alternatively, a member of the research team may request my permission to obtain access to my medical records for collection of follow-up information for the purposes of research and analysis.

	Consent of participant
In addition, I also give consent for my health information to be used for future research purposes:	☐ Yes ☐ No
I give permission for the research data collected about me in this study, to be linked with data from any other research study I participate in that is run as part of the Youth Mental Health Research Program led by Professor Ian Hickie.	☐ Yes ☐ No

Participant Consent Form V3.3 11/04/2023 Page 1 of 1

I would like to receive an overall summary of the results of this current study (via newsletter) once they are made available:	If yes, please provide email address:<br><br>_____________@___________________

Name of Participant (please print) ______________________________

Signature ______________________ Date ______________________

Name of Witness* to
Participant's Signature (please print) ______________________________

Signature ______________________ Date ______________________

* Witness is <u>not</u> to be the investigator, a member of the study team or their delegate. In the event that an interpreter is used, the interpreter may <u>not</u> act as a witness to the consent process. Witness must be 18 years or older.

<u>Declaration by Study Doctor/Senior Researcher†</u>

I have given a verbal explanation of the research project, its procedures and risks and I believe that the participant has understood that explanation.

Name of Study Doctor/
Senior Researcher† (please print) ______________________________

Signature ______________________ Date ______________________

† A senior member of the research team must provide the explanation of, and information concerning, the research project.

Note: All parties signing the consent section must date their own signature.

Hickie IB, *et al. BMJ Open* 2023; 13:e072082. doi: 10.1136/bmjopen-2023-072082

Form for Withdrawal of Participation

**Title**	A large-scale clinical effectiveness (health services) trial to determine whether personalised health care packages, combined with digitally-supported measurement-based care, improve functional outcomes in young people with mood disorders.
**Short Title**	EMPOWERED Trial.
**Protocol Number**	BMC-YMH-003-2018
**Project Sponsor**	The University of Sydney
**Principal Investigator**	Professor Ian B. Hickie
**Location**	Brain and Mind Centre, University of Sydney<br>Mind Plasticity, Surry Hills

Declaration by Participant

I wish to withdraw from participation in the above research project and understand that such withdrawal will not affect my routine treatment, my relationship with those treating me or my relationship with Brain and Mind Centre/Mind Plasticity.

Name of Participant (please print) ______________________

Signature ______________________ Date______________________

IF NECESSARY: Description of circumstances of withdrawal below (to be written by Study Doctor/Senior Researcher)

Declaration by Study Doctor/Senior Researcher†

I have given a verbal explanation of the implications of withdrawal from the research project and I believe that the participant has understood that explanation.

Name of Study Doctor/
Senior Researcher† (please print) ______________________

Hickie IB, *et al. BMJ Open* 2023; 13:e072082. doi: 10.1136/bmjopen-2023-072082

Signature ______________________________ Date ______________________________

† A senior member of the research team must provide the explanation of and information concerning withdrawal from the research project.

Note: All parties signing the consent section must date their own signature.
