# Supplementary material for: EMPOWERED trial: protocol for a randomised control trial of digitally supported, highly personalised and measurement-based care to improve functional outcomes in young people with mood disorders
Source: BMJ Open. 2023 Oct 11;13(10):e072082. doi: 10.1136/bmjopen-2023-072082 (PMC10583041; doi:10.1136/bmjopen-2023-072082)
Supplement: Author's manuscript [file bmjopen-2023-072082.draft_revisions.pdf]

BMJ Open is committed to open peer review. As part of this commitment we make the peer review history of every article we publish publicly available.

When an article is published we post the peer reviewers' comments and the authors' responses online. We also post the versions of the paper that were used during peer review. These are the versions that the peer review comments apply to.

The versions of the paper that follow are the versions that were submitted during the peer review process. They are not the versions of record or the final published versions. They should not be cited or distributed as the published version of this manuscript.

BMJ Open is an open access journal and the full, final, typeset and author-corrected version of record of the manuscript is available on our site with no access controls, subscription charges or pay-per-view fees (http://bmjopen.bmj.com).

If you have any questions on BMJ Open's open peer review process please email info.bmjopen@bmj.com

BMJ Open

The EMPOWERED trial: Protocol for a randomised control trial of digitally supported, highly personalised and measurement-based care to improve functional outcomes in young people with mood disorders

Journal:	*BMJ Open*
Manuscript ID	bmjopen-2023-072082
Article Type:	Protocol
Date Submitted by the Author:	02-Feb-2023
Complete List of Authors:	Hickie, Ian; The University of Sydney Brain and Mind Centre, Brain and Mind<br>Iorfino, Frank; The University of Sydney Brain and Mind Centre, Brain and Mind Centre<br>Rohleder, Cathrin; The University of Sydney Brain and Mind Centre<br>Song, Yun; The University of Sydney, Brain and Mind Centre<br>Nichles, Alissa; The University of Sydney, Brain and Mind Centre<br>Zmicerevska, Natalia; The University of Sydney, Brain and Mind Centre<br>Capon, William; The University of Sydney Brain and Mind Centre, Brain and Mind Centre<br>Guastella, Adam; Brain & Mind Research Institute, Clinical Research Unit<br>Leweke, F. Markus; The University of Sydney, Brain and Mind Centre; Ruprecht Karls University Heidelberg Faculty of Medicine Mannheim, Psychiatry and Psychotherapy, Central Institute of Mental Health<br>Scott, Jan; Newcastle University, Institute of Neuroscience<br>McGorry, Patrick; Oryegn Research Centre, University of Melbourne Australia<br>Mihalopoulos, Cathrine; Monash University, School of Public Health and Preventive Medicine<br>Killackey, Eoin; Orygen, The National Centre of Excellence in Youth Mental Health; University of Melbourne, Centre for Youth Mental Health<br>Chong, Min Kyung; The University of Sydney Brain and Mind Centre<br>McKenna, Sarah; The University of Sydney Brain and Mind Centre<br>Aji, Melissa; The University of Sydney Brain and Mind Centre<br>Gorban, Carla; The University of Sydney Brain and Mind Centre<br>Crouse, Jacob; The University of Sydney, Brain and Mind Centre<br>Koethe, Dagmar; The University of Sydney, Brain and Mind Centre<br>Battisti, Robert; Mind Plasticity<br>Hamilton, Blake; The University of Sydney, Brain and Mind Centre; headspace Camperdown<br>Lo, Alice; Mind Plasticity<br>Hackett, Maree; George Institute for Global Health, Faculty of Medicine, University of New South Wales; University of Central Lancashire, Faculty of Health and Wellbeing<br>Hermens, Daniel; Sunshine Coast Mind and Neuroscience Thompson Institute,<br>Consortia, Mind Plasticity; Mind Plasticity<br>Consortia, headspace Camperdown; headspace Camperdown

	Scott, Elizabeth; Brain & Mind Research Institute,
Keywords:	MENTAL HEALTH, PSYCHIATRY, Clinical trials < THERAPEUTICS

SCHOLARONE™
Manuscripts

The EMPOWERED trial: Protocol for a randomised control trial of digitally supported, highly personalised and measurement-based care to improve functional outcomes in young people with mood disorders

Ian B Hickie[1], Frank Iorfino[1*], Cathrin Rohleder[1], Yun Ju Christine Song[1], Alissa Nichles[1], Natalia Zmicerevska[1], William Capon[1], Adam J Guastella[1], F Markus Leweke[1,2], Jan Scott[3], Patrick McGorry[4,10], Cathrine Mihalopoulos[5], Eoin Killackey[4, 10], Min Kyung Chong[1], Sarah Mckenna[1], Melissa Aji[1], Carla Gorban[1], Jacob Crouse[1], Dagmar Koethe[1], Robert Battisti[6], Blake Hamilton[1,7], Alice Lo[6], Maree L Hackett[8], Daniel F Hermens[9], Mind Plasticity Consortia[6], headspace Camperdown Consortia[7] & Elizabeth M Scott[1]

[1] Brain and Mind Centre, The University of Sydney, Sydney, New South Wales, Australia
[2] Dept of Psychiatry and Psychotherapy, Central Institute of Mental Health, Medical Faculty Mannheim, Heidelberg University, Mannheim, Germany
[3] Newcastle University, Newcastle upon Tyne, England, United Kingdom
[4] Centre for Youth Mental Health, University of Melbourne, Melbourne, Victoria, Australia
[5] School of Public Health and Preventive Medicine, Monash University, Victoria, Australia
[6] Mind Plasticity, Sydney, New South Wales, Australia
[7] headspace Camperdown, Sydney, New South Wales, Australia
[8] The George Institute for Global Health, Sydney, New South Wales, Australia
[9] Thompson Institute, University of the Sunshine Coast, Birtinya, Queensland, Australia.
[10] Orygen, Melbourne, Australia.

Correspondence to:
Frank Iorfino, 94 Mallet Street Camperdown NSW 2050, frank.iorfino@sydney.edu.au

Sources of support: This study is supported by a National Health and Medical Research Council (NHMRC) Clinical Trials and Cohort Studies grant (grant number: 2001568).

Word Count: 3999/4,000

Number of figures and tables: 5 (3 figures, 2 tables)

For peer review only

Abstract (300 words)

Objectives

Many adolescents and young adults with emerging mood disorders do not achieve substantial improvements in education, employment, or social function after receiving standard youth mental health care. We have developed a new model of care referred to as 'highly personalised and measurement-based care' (HP&MBC). HP&MBC involves repeated assessment of multidimensional domains of morbidity to enable continuous and personalised clinical decision-making. Although measurement-based care is common in medical disease management, it is not standard practice in mental health. This clinical effectiveness trial tests whether HP&MBC, supported by continuous digital feedback, delivers better functional improvements than standard care and digital support.

Method and analysis

This controlled implementation trial (PROBE design) comprises a multi-site 24-month, assessor-blinded, follow-up study of 1500 individuals aged 15-25 years who present for mental health treatment. Eligible participants will be individually randomized (1:1) to 12 months of HP&MBC or standardised clinical care. The primary outcome measure is social and occupational functioning 12 months after trial entry, assessed by the Social and Occupational Functioning Assessment Scale (SOFAS). Clinical and social outcomes for all participants will be monitored for a further 12 months after cessation of active care.

Ethics and dissemination

This clinical trial has been reviewed and approved by the Human Research Ethics Committee of the Sydney Local Health District (HREC Approval Number: X22-0042 & 2022/ETH0072, Protocol ID: BMC-YMH-003-2018, protocol version: V.3, 03/08/2022). Research findings will be disseminated through peer-reviewed journals and presentations at scientific conferences and to user and advocacy groups. Participant data will be de-identified.

Trial registration number ACTRN12622000882729

For peer review only

Strengths and limitations of this study

Up to five short bullet points, no longer than one sentence each, that relate specifically to the methods.

1. To our knowledge, this is the first large scale effectiveness trial that tests whether early intervention and secondary prevention deliver substantive improvements in functional outcomes for young people with major mood disorders.
2. The trial sample will be large, and the use of minimal eligibility criteria maximises the generalisability of these findings to other youth mental health settings
3. The trial introduces new service roles (i.e., 'digital navigator', 'clinical facilitator') to help clinicians and clients to access the optimal package of interventions.
4. Standard care packages are delivered in the same setting and by the same health professional as the intervention group. So cross-over effects may attenuate between groups differences.
5. The availability and access to specific interventions needed to deliver enhanced care to those in the intervention group may be variable across different trial sites.

For peer review only

Introduction

There has been increasing recognition of the premature death and persistent disability attributable to the major mental disorders[1, 2]. The largest proportion of this excessive morbidity is attributable to mood disorders, reflecting their early age of onset, high population prevalence, chronicity and comorbidity [3]. While significant investments have been made in youth mental health services internationally, there is a lack of substantive evidence for which models of care are optimal for improving illness outcomes.

Mood disorders place young people at risk of prolonged socioeconomic difficulties, even when their mental ill-health subsides[8, 9]. Our work has identified that up to two-thirds of young people in youth mental health services experience poor longer-term functional outcomes [10, 11]. Current evidence suggests that youth mental health services primarily benefit those in the earlier stages of illness and that while brief psychological interventions are effective for reducing psychological distress, they only marginally improve functioning[12]. Further, current models of care do not appear to be well suited to those with comorbidities mixed or attenuated symptomatology, or social and occupational complexity. Most treatment plans are focused narrowly on limited treatment choices or 'steps' for discrete disorders. They are based on average population effects or clinical experience [13-21], and are often inaccurate and inconsistent.

The differentiation of young people with 'reasonable/good' from 'impaired/poor' functioning at presentation is a key factor to be considered (alongside other clinical variables) to determine the need for highly personalised care with the appropriate type, intensity, sequence, and duration of multidisciplinary interventions. This approach aligns with optimal models of mental health care and should be a key component of youth mental health service provision[24]. Yet, the evidence-base for health service models that guide personalised interventions for young people with mood disorders is sparse[5, 25-27]. Furthermore, it is not standard practice to use measurement-based care (MBC) for the monitoring of symptoms and functioning to drive continuous and personalised clinical decision making[28-32]. Highly personalised and measurement-based care is a core component of the chronic care model and supports better-informed clinical decisions[33-39]. Despite good evidence for its effectiveness and its customary use in physical disease management[36, 37], it remains largely absent from youth mental health care[13, 40].

Objectives of the study

The primary objective of this large-scale clinical effectiveness trial is to assess the effectiveness of 12 months of intensive, personally tailored, assertive care (the digitally supported HP&MBC package), compared with digitally-supported standard clinical care. We will test whether the HP&MBC package results in a greater improvement of social and occupational functioning compared to standard clinical care. The secondary objective is to assess the mental health status of all participants 12 months after the enhanced and standard care interventions.

For peer review only - http://bmjopen.bmj.com/site/about/guidelines.xhtml

We hypothesise that while the standard care packages will be an improved offering (through greater standardisation of assessment and access to digital technology), the HP&MBC treatment packages will be superior by implementing continuous and proactive monitoring and care coordination using digital technologies and providing extensive feedback to the clinical service, the treating clinician, the young person and their family or carer[41].

Methods and analysis

Study design and setting

This large-scale, prospective study aims to enrol 1500 mental health treatment seeking young people with mood disorders. The trial was designed with the aid of young people with lived experience of mental illness and comprises a 24-month (12 months active treatment, 12 months additional follow-up), multi-site, two-arm (HP&MBC care package vs standardised clinical care), randomised (1:1), blinded outcome assessor, controlled implementation trial (PROBE design). The trial will be conducted at the Brain and Mind Centre (The University of Sydney, Australia) and affiliated youth centres that focus on treating young people with mental illness. As noted below, prior to commencing the RCT, there will be a pre-trial phase (Figure 1).

Pre-trial phase

The study includes a pre-trial phase to allow the digital technology platform to be introduced to the clinical teams and integrated into the service procedures. This period will be used to work through any implementation issues prior to commencing the RCT. Also, it permits collection of pre-trial data from each clinical service, including an audit of outcomes in routine clinical practice (e.g., rates of improvement or deterioration in social and occupational function in non-trial clinical cohorts).

RCT and follow up phase (~24 months)

After the pre-trial phase, the RCT phase of the study will commence (expected in early 2023). Participation in the trial will be 24 months following enrolment (baseline assessment), including 12 months of active treatment and 12 months additional follow-up. Five independent assessments will be conducted: at baseline, three months, six months, 12 months, and 24 months.

Patient and public involvement

Young people with lived experience of mental illness were invited to participate in the study design through consultation with the Brain and Mind Centre Lived Experience Working Group. The working group consists of culturally and linguistically diverse young people aged 16-30 years old. The principles underpinning the trial, and the name for this trial 'EMPOWERED', were identified by young people with lived experience (textbox 1).

For peer review only

Study Population

The study focuses on young people seeking help for psychological distress and presenting with early stage mood syndromes, characterised not only by the mix of anxiety or depressive symptoms, and their impact on function, but also according to stage of illness criteria (Stage 1a – non-specific anxiety and depressive syndromes), attenuated syndromes (Stage 1b) or first full-threshold, major and discrete mood syndrome (Stage 2)[42]. Recruitment is also based on the presentation to care and existing functional impairment. This approach is consistent with the National Institute of Mental Health recommendations for conducting more integrative clinical research[18]. Approximately 10,000 individuals aged 15-25 years present to the Brain and Mind Centre and affiliated youth centres per year. We expect that 3000 individuals will meet the inclusion criteria and that about 50% of the eligible individuals will consent. In total, 1500 young people will be included (750 allocated to the active 12-month care package and 750 allocated to the standardised care package).

Textbox 1. The EMPOWERED trial principles

1. **E**ducate – To educate young people, and their families and carers, on the potential usefulness of technology, and how routine monitoring can give them a greater say in their care journey.
2. **M**easurement-based – To improve continuous and real-time measurement of young people's symptoms and functioning, and longer-term outcomes, so that they can receive more effective care.
3. **P**ersonalised – Ensuring that treatment is personalised, so that the complexity of young people's needs are recognised, documented, acted on and preserved over the care journey.
4. **O**penness – Improving open communication between young people, their families and carers, and clinicians by making everyone more informed about progress in care.
5. **W**ork collaboratively – Helping clinicians and young people to work collaboratively to create and respond to treatment goals by facilitating treatment monitoring, emphasizing functional recovery, and allowing young people to focus on assessment domains that matter most to them.
6. **E**ngage – Increasing young people's engagement in care planning, by putting information about their mental health into their own hands.
7. **R**ecovery – Earlier recovery through improved clinical and functional assessment, and actively monitoring social, education and employment outcomes, to ensure that young people receive earlier and more personalised care.
8. **E**nhanced **D**igitally – Leverage the advanced capabilities of digital technologies to facilitate the assessment, monitoring and management of mental health problems, and support shared and informed decision making.

Inclusion and exclusion criteria

Participation in this study will be offered to adolescents and young adults aged 15- 25-years seeking help for psychological distress and classified as Stage 1a, 1b or 2. The participants must have an initial Social and Occupational Functioning Assessment Score (SOFAS) of ≤ 70[43], indicating impaired social and occupational functioning. Young people with a lifetime diagnosis of a full-threshold psychotic or bipolar I disorder, or alcohol or other substance dependence, will be excluded. Additional exclusion criteria include acute suicidal or aggressive behaviour requiring alternative care or a depressive syndrome secondary to a primary medical condition. Young people who have a clinically evident intellectual disability (IQ<70 as per

medical history review) will be excluded due to the likely difficulty in completing the assessments.

Study course and procedures

The clinical trial comprises 12-months active treatment and 12-months follow-up phase, i.e., each subject will be followed for two years, whereby five blinded independent assessment visits will take place (Figure 2).

Individuals referred to the trial will be contacted by a research team member who will provide information about the study and conduct a preliminary assessment of the inclusion and exclusion criteria. Potential participants interested in taking part in the study will then be provided with a copy of the participant information statement, and an appointment will be scheduled for an enrolment visit. During the enrolment/baseline visit (Visit 0), the study will be explained in lay terms and any questions will be answered. Following informed consent, participants will be given relevant assessments to confirm that they meet the inclusion criteria.

Participants who meet the inclusion criteria will be randomised to one of the two treatment arms using a 1:1 individual person randomisation algorithm taking age, gender and treatment centre as stratification factors into account. The care packages will be delivered within the first 12 months of the study by clinicians operating within each service. During this study phase, three study visits will take place: (i) Visit 1 (3 months after trial entry); (ii) Visit 2 (6 months after trial entry); and (iii) Visit 3 (12 months after trial entry). A follow-up visit (Visit 4) will be conducted 12 months after completion of the care packages (Figure 2). All follow up assessments will be carried out by blinded-independent assessors from the research team.

Care Packages

To coordinate ongoing clinical care and functional recovery, real-time data feedback will be provided in both treatment arms using health information technology. As demonstrated in our longitudinal studies[44-46], those with attenuated syndromes often receive only brief interventions (six or fewer sessions of psychological support) and exit services with residual high levels of impairment. There is a fundamental mismatch between the time course of impairment (typically well-established by the time the young person presents to clinical care) and the brief clinical interventions provided by early intervention services. Therefore, we now use personalised technologies to tailor, plan, and track the relationships between clinical care delivery (and sequencing) and functional recovery strategies. The real-time data feedback will support optimal combinations of indirect and direct intervention strategies until the young person achieves: (i) syndromal remission and risk reduction and (ii) social and occupational recovery. This real-time data feedback will be supported by the Innowell Platform[47].

The digitally supported HP&MBC care package represents an intensive, personalised and assertive treatment package. It builds on the usual processes provided by the services at each

For peer review only

centre, including systematic assessment and allocation of clinical care within multidisciplinary team environments. The HP&MBC care package uses two key streams, namely (a) the therapeutic power of active and continuous feedback with regards to illness type, course, response to interventions and social and economic impact of care; and (b) the capacity of new assessment and monitoring techniques to tailor treatment options – with the standardisation of those stepped-care options into an on-going and proactive shared care plan.

The HP&MBC enhanced care package includes:

(i) Initial digital assessment covering the domains of symptoms, social and occupational functioning, self-harm and suicidal thoughts and behaviours (STBs), physical health and alcohol and other substance misuse;
(ii) Feedback of the initial 'dashboard' of results to the user of care and family members, clinical services and the principal treating clinician (Figure 3);
(iii) Continuous outcome monitoring and feedback – Regular review of 'dashboard' to the user of care and family members, clinical services, and principal treating clinician (monthly for first 12-months, may vary based on individual needs);
(iv) More detailed online, clinical, neuropsychological and lab-based testing as recommended by digital or clinical protocols, including use of specific individual monitoring devices (e.g., wearable activity monitors, mood monitors) to inform broad diagnostic categorisation and then assign a more specific series of highly personalised treatment options;
(v) Determination of indicative sub-type of depressive syndrome by incorporation of clinical factors and life course, to link to specific intervention strategies;
(vi) Utilisation of online shared care planning by the user of care and family members, clinical services, and principal treating clinician;

Active and continuous feedback will guide evidence-based decision making related to treatment plans as it supports the choice of optimal combinations of interventions. The measurement-based feedback will help detect unmet needs, increase the likelihood that clinicians identify young persons who are non-responsive to treatment, and facilitate the process to adjust the plan of care to improve young person outcomes.

The standard care package builds on the usual service systems (largely Medicare-funded psychological care), including systematic assessment and allocation of clinical care within multidisciplinary team environments.

The standardised care package includes:

(i) Initial digital assessment covering the domains of symptoms, social and occupational function, self-harm, and STBs, physical health and alcohol and other substance misuse;
(ii) Feedback of the initial 'dashboard' of results to the user and treating clinician at baseline;

For peer review only

(iii) Provision of standard multidisciplinary care options and ongoing access to other relevant psychological and pharmacological options;

In this study, the following targeted therapies (over and above standard psychological care), which have been shown in various studies to have beneficial effects[48-57], are of particular relevance:

(i) *Social and Functional Recovery Therapies:* Interventions that target social recovery include direct support to return to work, re-engaging in education or training, and social skills training to reduce isolation and improve relationships with peers and family. Key components of these interventions include setting meaningful recovery goals, establishing the external resources to support recovery, and using outreach graded behavioural experiments to re-establish functioning.

(ii) *Circadian Interventions:* Pharmacological (e.g., agomelatine, brexpiprazole), physical (e.g., light therapy) or behavioural interventions (e.g., sleep-wake rescheduling) that target dysregulation of sleep-wake behaviours and biological circadian rhythms.

(iii) *Cognitive-Behaviour Therapies (CBT) and Social Therapies Groups:* CBT teaches the individual to link their feelings, thoughts, and patterns of behaviours to reduce psychological distress. A greater focus on social cognition training may be needed for those with social cognitive impairment.

(iv) *Dialectic Behaviour Therapy (DBT):* DBT is a modified version of CBT designed to treat symptoms often associated with emotional dysregulation and poor distress tolerance such as self-harm, suicidal behaviour, and substance use. The emphasis is on moving away from harmful coping behaviours and incorporates mindfulness, distress tolerance, emotional regulation, and interpersonal effectiveness strategies.

(v) *Healthy lifestyle and cardiometabolic health targeted treatments:* The World Health Organisation guidelines recommend that lifestyle behavioural interventions be considered the first-line treatments for managing physical health (including cardiometabolic health) for those with severe mental illness. Psychoeducational interventions focusing on healthy lifestyle habits including diet, physical activity and sleep practices have been shown to ameliorate both the physical and mental health concerns of young people with psychiatric disorders.

Whilst these therapies will be available to participants in both treatment arms, those in the HP&MBC treatment group will be actively referred to the specific optimal treatment program/s based on the outcomes of the continuous assessment data that will be made available to the participant and their treating clinician. In addition to the targeted therapies mentioned above, additional relevant therapies may be introduced over the course of the study as the clinical needs of participants become apparent.

Service roles

For peer review only

Two additional service roles will be employed for the trial (Table 1). The first is a 'clinical facilitator' who is an independent clinician focused on ensuring optimal uptake of the HP&MBC by the treating clinicians. This will be achieved by working collaboratively with clinicians with the aim of reducing the additional tasks associated with enhanced and rapid communication, tracking, and interpreting and actioning feedback. The second role is a 'digital navigator' who will operate for participants across both arms of the trial. The primary focus of the role is to provide peer support for young people to motivate them to provide outcome data; regularly remind them of the purpose of collecting data and how it can improve their treatment journey; help the young person, carer and clinician to address technical issues; and provide guidance about useful e-tools to be used in treatment.

For peer review only

Table 1. Role description of the facilitator team.

Description of Roles	Tasks	Examples
Clinical Facilitator<br>*Only available for participants intervention arm*<br><br>The purpose of this role is to facilitate the use of the HP&MBC by clinicians. This is achieved by working collaboratively with clinicians with the aim of reducing burden associated with communication, tracking, and interpreting and actioning feedback. The main responsibilities of this role include:<br>a) Assisting clinicians to review and aid identification of any domains of concern (e.g. increased risk or decreased social support);<br>b) Providing logistical support in making referrals for clients<br><br>This role does not have any clinical responsibility towards clients as this is a support role.	• Promote and assist with the use of routine client feedback to inform personalised treatment options<br>• Reduce time burden for clinicians by monitoring client progress using technology and alerting clinicians if significant deteriorations/risk arise<br>• Performing administrative tasks to facilitate referrals, and identify where appropriate treatment options recommended by the youth model<br>• Regularly assess with clinicians how client feedback has been used in sessions to inform treatment<br>• Develop a good understanding of referral options in the relevant area including community organisations, schools, public health services, online services and apps, etc<br>• Assist with identifying appropriate care options and help with the logistics of the organisation of clinical care.	**SOFAS deterioration**<br>Clinical facilitator (CF) notes that patient X's SOFAS has deteriorated ten points since their last report one month ago. CF communicates with X's psychiatrist, using their preferred communication method, letting them know that there has been a deterioration. Psychiatrist notifies CF that they have commenced a new course of treatment at their last appointment two weeks ago and will continue monitoring their symptoms. CF also communicates with psychologist to let them know about deterioration and notes that psychiatrist has changed medication recently. Psychologist notes that client X has recently begun exposure exercises in their weekly therapy sessions that they are finding highly distressing.<br><br>One month later, the client reports further deterioration to SOFAS and that they have experienced an increase in passive suicidal ideation. CF communicates this to the psychiatrist and psychologist. Psychiatrist requests DBT and CF facilitates meeting between psychologist and psychiatrist to discuss options.<br><br>CF also contacts three local community and public health services that offer DBT programs and finds that Cremorne Health Centre has a spot available for client X. CF passes this information to psychiatrist to make referral.
Digital Navigator<br>*Available to participants in both arms of the trial*<br><br>The primary focus of the role is to:	• Troubleshoot any issues related to technology for clients, caregivers and clinicians<br>• Remind clients to complete	**Enrolment of a new participant**<br>Client X newly joined the trial. The DN will organise a brief meeting with the client to introduce Innowell and to educate them on

For peer review only

a) provide peer support for clients to motivate them to provide outcome data - regularly remind the clients of the purpose of collecting data and how it can improve their treatment journey;<br>b) help client, carer and clinician to address other technical issues;<br>c) provide guidance about useful etools (online resources and apps) to be used in treatment;	Innowell questionnaires. Routinely follow up with clients, through their preferred method (e.g. text, email or face to face) to ensure regular data collection<br>• Research evidence-based etools that clinicians can confidently use as part of treatment	the purpose of using the platform and its potential benefits.<br><br>After 1 month, DN follows up with client X to collect feedback about their experience of Innowell and whether regular reporting about their symptoms has been used by clinicians to inform treatment.<br><br>Client X states that they liked how their functional scores were discussed during the session but wished that their physical status was addressed. DN relays the feedback and suggest an app that can monitor client physical status to CF. CF alerts clinicians about client X's physical scores and promotes active response.

Assessments

A series of standardised clinical assessments will be conducted at the enrolment visit (Visit 0) to assess inclusion and exclusion criteria (Table 2), including:

1. Structured Clinical Interview for DSM-5 to assess the presence of mental health and substance use disorders.
2. A framework for clinical staging[48, 58] will be applied to assess illness severity and differentiate those in the earliest phases with non-specific clinical presentations (stages 1a 'seeking help') from those at greater-risk with more specific, sub-threshold presentations (stage 1b 'attenuated syndromes') and those who have reached a threshold for a progressive or recurrent disorder meeting diagnostic criteria (stage 2, 3, or 4).
3. Social and Occupational Functioning Assessment Scale (SOFAS) to record the clinician's judgement of overall social and occupational function.
4. A mental risk assessment to assess acute suicidal behaviour.

As summarised in Table 2, individuals who fulfil all inclusion and exclusion criteria will undergo additional clinician/researcher-administered baseline assessments evaluating depressive symptomatology, personal social performance, and self-report questionnaires will be provided to collect information regarding the quality of life, self-harm, suicidal thoughts and behaviours, alcohol and substance use, and physical health. Furthermore, blood will be collected to assess metabolic, inflammatory and standard blood markers.

While social and occupational functioning, illness severity, and depressive symptoms will be assessed at every subsequent visit (Visits 1-4), the structured clinical interview will only be

For peer review only

repeated at the end of the active treatment phase (12 months after trial entry, Visit 3; and 24 months after trial entry, Visit 4). Self-report questionnaires will be provided at each visit during the active treatment phase (visits 1-3). Blood samples will be collected at baseline, 6 months, 12 months and 24 months after trial entry (i.e., at visits 0, 2, 3 and 4) to monitor changes in metabolic, inflammatory and standard blood markers.

Resource use that will also be used to estimate costs will be measured using two main procedures:

1. Participants will be asked for access to administrative data sets including the Medicare Benefits Schedule (MBS) and Pharmaceutical Benefits Schedule (PBS) data for the duration of the study.

2. The resource use questionnaire, used in multiple mental health economic evaluations, which captures the broad range of health and welfare services used by participants and is complementary to any administrative data also included in the evaluation [59, 60].

Micro-costing techniques will be used to assess the costs of the intervention. Standardised economic evaluation techniques including incremental analysis of mean differences using generalised linear models, and bootstrapping to determine confidence intervals will also be used. Lifetime and population cost-effectiveness will be also determined using economic modelling techniques.

Table 2: Overview of research assessments. Abbreviations: AUDIT-C, Alcohol Use Disorders Identification Test – Consumption; BMI, Body Mass Index; B-NSSI-AT, Brief Non-suicidal Self-injury Assessment Tool; CAARMS (7.3), Comprehensive Assessment of At-Risk Mental States – item 7.3; C-SSRS, Columbia-Suicide Severity Rating Scale; DSM-5, Diagnostic and Statistical Manual of Mental Disorders, 5th edition; IPAQ, International Physical Activity Questionnaire – short version; PSP, Personal and Social Performance Scale; QIDS-C, Quick Inventory of Depressive Symptomatology – Clinician-rated; ReQoL-10, Recovering Quality of Life Questionnaire (10-item version); SIDAS, Suicidal Ideation Attributes Scale; SOFAS, Social and Occupational Functioning Assessment Scale; WHO-ASSIST, World Health Organisation Alcohol, Smoking and Substance Involvement Screening Test (version 3.1).

**Domain**	**Assessment**	**Administration**	**Time points (Months)**				
			**0**	**3**	**6**	**12**	**24**
**Clinical Diagnosis**	Structured Clinical Interview to assess for DSM-5 Mental Health and Substance Use Disorders	Researcher administered	✓			✓	
**Acute suicidal and aggressive behaviour (exclusion criteria)**	CAARMS (7.3 and 5.4)	Researcher administered	✓				

For peer review only

**Social and occupational functioning**	SOFAS	Researcher administered	✓	✓	✓	✓	✓
**Social and occupational functioning**	PSP	Researcher administered	✓	✓	✓	✓	✓
**Depressive Symptoms**	QIDS-C	Researcher administered	✓	✓	✓	✓	✓
**Illness severity**	Clinical staging	Researcher administered	✓	✓	✓	✓	✓
**Quality of life**	ReQoL-10	Self-report	✓	✓	✓	✓	✓
**Self-harm / suicidal thoughts and behaviours**	SIDAS / adaptation of the C-SSRS / B-NSS-AT	Self-report	✓	✓	✓	✓	
**Alcohol and substance use**	AUDIT-C – Alcohol use<br>WHO-ASSIST – Alcohol and other substance use	Self-report	✓	✓	✓	✓	
**Physical health**	Height / weight / waist / BMI	Self-report	✓	✓	✓	✓	
**Physical health**	IPAQ (physical activity)	Self-report	✓	✓	✓	✓	
**Physical health**	Metabolic, inflammatory & standard clinical bloods	Researcher administered	✓		✓	✓	
**Resource Use**	Resource Use Questionnaire	Self-report	✓	✓	✓	✓	✓

Primary and secondary outcomes

Primary efficacy endpoint:

- Changes in social and occupational function from baseline to 12 months, as assessed by the SOFAS.

Key secondary endpoints:

- Change from baseline in self-harm, suicidal thoughts and behaviours (B-NSSI-AT, SIDAS, C-SSRS)
- Change from baseline in depressive symptoms (QIDS)
- Change from baseline in quality of life (ReQoL)[61, 62]
- Change from baseline in alcohol and substance use (WHO-ASSIST, AUDIT-C)
- Change from baseline in physical health (IPAQ, height, weight, waist)
- Change from baseline in metabolic, inflammatory and standard blood measures (metabolic and inflammatory markers)
- Resource use as well as lifetime and population cost-effectiveness.
- Costs of the treatment packages based on detailed economic evaluation.

Sample size calculation

This trial seeks to recruit 1,500 young people, with 750 allocated to active 12-month intervention and 750 to standard clinical care. We anticipate an attrition rate of approximately 10-20% over short-term follow up (first 12 months) and up to 30% over the longer-term follow-up (at 24 months). Therefore, we would expect 1350 participants at six months follow-

For peer review only

up (675 in each arm), 1200 participants at 12 months follow-up (600 in each arm) and 1050 participants at two years follow-up (525 in each arm). Assuming that we have at least 434 young people at the two-year follow-up time point, for the primary outcome analysis only, and conservatively assuming a small effect size difference of 0.2 in favour of those young people receiving the active intervention, α=0.05, we have 95% power. For categorical secondary analyses, a small effect size of 0.2, α=0.05, power=95%, sample size at two-year follow-up is 325 participants. There are also embedded sub-groups for secondary analyses (e.g., by baseline suicidal acts, depressive sub-type, alcohol or other substance misuse and baseline SOFAS bands). For these subgroups, assuming that we have at least 195 young people at the two-year follow-up time point, for the primary outcome analysis only, and conservatively assuming a medium effect size difference of 0.3 in favour of those young people receiving the active intervention, α=0.05, we have 95% power. For categorical secondary analyses, a medium effect size of 0.3, α=0.05, power=95%, sample size at two-year follow-up is 144 participants.

Data analysis plan

The primary outcome will be analysed using a repeated-measure linear mixed model including all available SOFAS scores measured at months 3, 6, 12 and 24. Fixed effects will include the randomised group, visit as a categorical variable and the interaction between group and visit. The baseline SOFAS score will be included as a covariate alongside sex, age and site (stratification variables). To account for correlations between repeated measures, a random patient intercept will be included. In case of convergence issues with the inclusion of the random effect, we will replace the random effect with a repeated effect assuming a compound symmetry covariance structure. This model will be used to derive the effect of the intervention at 12 months, expressed as the adjusted mean difference and its 95% confidence interval. The effect of the intervention at other timepoints will be estimated using a similar approach.

Secondary outcomes will be analysed using a similar approach. For binary outcomes, logistic regression (binomial distribution with logit link) will be used in place of linear regression. The effect of the intervention will be estimated as the odds ratio and 95% confidence interval and converted to an absolute risk difference using the Hummel and Wiseman method[63]. Given that linear mixed models use all data available and make valid inference under the assumption that data is missing at random, the primary analysis will not impute missing data; however, sensitivity analyses will be conducted to assess the robustness of the results under different assumptions about the missing data mechanism. A detailed statistical analysis plan including mock tables will be developed prior to unblinding and database lock.

The economic evaluation of the HP&MBC package is critical to translate this research into practice. It will comprise both a "within-trial" design whereby the individual level costs and outcomes of the two groups (HP&MBC and Standard Care packages) will be included in the evaluation over the duration of the trial. A modelled evaluation will be undertaken to capture full costs and consequences of HP&MBC, using the results of this trial and the broader

For peer review only - http://bmjopen.bmj.com/site/about/guidelines.xhtml

epidemiological literature to estimate likely longer term health gains, cost impacts and scale up costs at the population level. The calculation of quality-adjusted life years (QALYs) will be done, thus enabling a cost-utility analysis to be undertaken. Cost-utility analyses are useful to decision-makers as they are associated with inherent value for money connotations. Detailed costing of the HP&MBC approach along with how it has been implemented within each site will be undertaken using information from the researchers, clinical staff, and budgetary personnel.

Ethics and dissemination

The study will be performed according to the Declaration of Helsinki (2008) and the International Conference on Harmonisation - Good Clinical Practice (ICH-GCP) and has been reviewed and approved by the Human Research Ethics Committee (HREC) of the Sydney Local Health District (HREC Approval Number: X22-0042 & 2022/ETH0072, Protocol ID: BMC-YMH-003-2018, protocol version: V.3, 03/08/2022). The study has been registered in the Australian New Zealand Clinical Trial Registry (ACTRN12622000882729). The results of this study will be disseminated as widely as possible into the scientific and broader community, including include publication in peer-reviewed journals, scholarly book chapters, presentation at conferences and publication in conference proceedings.

Contributorship statement

IBH conceived the research idea and is the principal investigator. FI, AN, YJCS and NZ contributed to the study design and conception. CR, AN, YJCS and NZ wrote the study protocol with input from IBH, FI, WC, AJG, FML, JS, PM, CM, EK, MKC, SM, MA, CG, JC, DK, RB, BH, AL, MLH, DFH, MPC, HCC and ES. FI and CR wrote the manuscript with input from IBH, AN, NZ, YJCS, NZ, WC, AJG, FML, JS, PM, CM, EK, MKC, SM, MA, CG, JC, DK, RB, BH, AL, MLH, DFH and ES.

Acknowledgements

We are deeply indebted to all clinicians and health professionals at the participating centres who contribute to this clinical trial. Particularly to the Mind Plasticity Consortia which includes: Mark Yim, Bradley Whitwell and Alison Crowley; and the headspace Camperdown Consortia which includes Donna Fowler, Elana Volanakis, Vicki Papageorgopoulos, Matilda Russel and Elizabeth Phung.

Funding

This trial is an investigator-initiated trial funded by the NHMRC – 2020 Clinical Trials and Cohort Studies, Application ID: 2001568. Frank Iorfino is supported by the Bill and Patricia Ritchie Foundation Fellowship. Jacob Crouse is supported by a NHMRC Emerging Leadership Fellowship (GNT2008197).

Competing interests

For peer review only

A/Prof Elizabeth Scott is the Medical Director, Young Adult Mental Health Unit, St Vincent's Hospital Darlinghurst, Discipline Leader of Adult Mental Health, School of Medicine, University of Notre Dame, Research Affiliate, The University of Sydney and Consultant Psychiatrist. She has received honoraria for educational seminars related to the clinical management of depressive disorders supported by Servier and Eli-Lilly pharmaceuticals. She has participated in a national advisory board for the antidepressant compound Pristiq, manufactured by Pfizer. She was the National Coordinator of an antidepressant trial sponsored by Servier.

Professor Jan Scott is a visiting professor at Diderot University, the Norwegian University of Science and Technology, Swinburne University of Technology and The University of Sydney and a 'Science without Borders' fellow (Brazil). She has received grant funding from the UK Medical Research Council and from the UK Research for Patient Benefit programme; she declares no financial or other conflict of interests in relation to the topics addressed in this paper.

Professor Ian Hickie is the Co-Director, Health and Policy at the Brain and Mind Centre (BMC) University of Sydney, Australia. The BMC operates an early-intervention youth services at Camperdown under contract to headspace. Professor Hickie has previously led community-based and pharmaceutical industry-supported (Wyeth, Eli Lily, Servier, Pfizer, AstraZeneca) projects focused on the identification and better management of anxiety and depression. He is the Chief Scientific Advisor to, and a 3.2% equity shareholder in, Innowell Pty Ltd. Innowell was formed by the University of Sydney (45% equity) and PwC (Australia; 45% equity) to deliver the $30 M Australian Government-funded Project Synergy (2017-20) and to lead transformation of mental health services internationally through the use of innovative technologies.

All other authors declare no conflict of interest.

For peer review only

References

1. Bloom, D., et al., *The global economic burden of non-communicable diseases*. 2012, Program on the Global Demography of Aging.
2. Gustavsson, A., et al., *Cost of disorders of the brain in Europe 2010.* European Neuropsychopharmacology, 2011. **21**(10): p. 718-79.
3. Lopez, A.D., et al., *Global and regional burden of disease and risk factors, 2001: Systematic analysis of population health data.* The Lancet, 2006. **367**(9524): p. 1747-1757.
4. Insel, T.R., *The arrival of preemptive psychiatry.* Early Interv Psychiatry, 2007. **1**(1): p. 5-6.
5. Insel, T.R., *Translating scientific opportunity into public health impact: A strategic plan for research on mental illness.* Archives of General Psychiatry, 2009. **66**(2): p. 128-133.
6. Scott, E.M., et al., *Targeted primary care-based mental health services for young Australians.* Med J Aust, 2012. **196**(2): p. 136-40.
7. Hickie, I.B., et al., *Applying clinical staging to young people who present for mental health care.* Early Intervention in Psychiatry, 2013. **7**(1): p. 31-43.
8. Merikangas, K.R., et al., *Lifetime prevalence of mental disorders in U.S. adolescents: results from the National Comorbidity Survey Replication--Adolescent Supplement (NCS-A).* J Am Acad Child Adolesc Psychiatry, 2010. **49**(10): p. 980-9.
9. Copeland, W.E., et al., *Adult Functional Outcomes of Common Childhood Psychiatric Problems: A Prospective, Longitudinal Study.* JAMA Psychiatry, 2015. **72**(9): p. 892-9.
10. Iorfino, F., et al., *Social and occupational outcomes for young people who attend early intervention mental health services: a longitudinal study.* Med J Aust, 2022. **216**(2): p. 87-93.
11. Iorfino, F., et al., *Delineating the trajectories of social and occupational functioning of young people attending early intervention mental health services in Australia: A longitudinal study.* BMJ Open, 2018. **8**(3): p. e020678.
12. Cross, S.P., D.F. Hermens, and I.B. Hickie, *Treatment patterns and short-term outcomes in an early intervention youth mental health service.* Early Intervention in Psychiatry, 2016. **10**(1): p. 88-97.
13. Hatfield, D., et al., *Do we know when our clients get worse? An investigation of therapists' ability to detect negative client change.* Clinical Psychology & Psychotherapy: An International Journal of Theory & Practice, 2010. **17**(1): p. 25-32.
14. Walfish, S., et al., *An investigation of self-assessment bias in mental health providers.* Psychological Reports, 2012. **110**(2): p. 639-644.
15. Scott, J., et al., *Pathways to depression by age 16 years: examining trajectories for self-reported psychological and somatic phenotypes across adolescence.* Journal of Affective Disorders, 2017.
16. Hansell, N.K., et al., *Genetic co-morbidity between neuroticism, anxiety/depression and somatic distress in a population sample of adolescent and young adult twins.* Psychological Medicine, 2012. **42**(6): p. 1249-1260.
17. Scott, J., et al., *Clinical staging in psychiatry: a cross-cutting model of diagnosis with heuristic and practical value.* The British Journal of Psychiatry, 2013. **202**(4): p. 243-245.
18. Cuthbert, B.N., *The RDoC framework: facilitating transition from ICD/DSM to dimensional approaches that integrate neuroscience and psychopathology.* World Psychiatry, 2014. **13**(1): p. 28-35.

For peer review only

19. Casey, B., et al., *DSM-5 and RDoC: progress in psychiatry research?* Nature Reviews Neuroscience, 2013. **14**(11): p. 810.
20. Ægisdóttir, S., et al., *The meta-analysis of clinical judgment project: Fifty-six years of accumulated research on clinical versus statistical prediction.* The Counseling Psychologist, 2006. **34**(3): p. 341-382.
21. Woodford, R., et al., *Accuracy of clinician predictions of future self-harm: a systematic review and meta-analysis of predictive studies.* Suicide and Life-Threatening Behavior, 2019. **49**(1): p. 23-40.
22. Richards, D.A., et al., *Delivering stepped care: an analysis of implementation in routine practice.* Implementation Science, 2012. **7**(1): p. 3.
23. Jorm, A.F., et al., *Has increased provision of treatment reduced the prevalence of common mental disorders? Review of the evidence from four countries.* World Psychiatry, 2017. **16**(1): p. 90-99.
24. Hickie, I.B., et al., *Right care, first time: a highly personalised and measurement-based care model to manage youth mental health.* Medical Journal of Australia, 2019. **211**: p. S3-S46.
25. Iorfino, F., et al., *Multidimensional outcomes in youth mental health care: what matters and why?* MEDICAL JOURNAL OF AUSTRALIA, 2019. **211**: p. S4-S11.
26. Insel, T.R., *The arrival of preemptive psychiatry.* Early Intervention in Psychiatry, 2007. **1**(1): p. 5-6.
27. Scott, E.M., et al., *Targeted primary care-based mental health services for young Australians.* Medical Journal of Australia, 2012. **196**(2): p. 136-140.
28. Harding, K.J., et al., *Measurement-based care in psychiatric practice: a policy framework for implementation.* J Clin Psychiatry, 2011. **72**(8): p. 1136-43.
29. Goldberg, S.B., et al., *Measuring Psychiatric Symptoms Remotely: a Systematic Review of Remote Measurement-Based Care.* Curr Psychiatry Rep, 2018. **20**(10): p. 81.
30. Fortney, J.C., et al., *A Tipping Point for Measurement-Based Care.* Psychiatric Services, 2017. **68**(2): p. 179-188.
31. Waldrop, J. and T.M. McGuinness, *Measurement-Based Care in Psychiatry.* J Psychosoc Nurs Ment Health Serv, 2017. **55**(11): p. 30-35.
32. Hickie, I.B., *The role of new technologies in monitoring the evolution of psychopathology and providing measurement-based care in young people.* World Psychiatry, 2020. **19**(1): p. 38.
33. Lewis, C.C., et al., *Implementing measurement-based care (iMBC) for depression in community mental health: a dynamic cluster randomized trial study protocol.* Implementation Science, 2015. **10**(1): p. 127.
34. Scott, K. and C.C. Lewis, *Using measurement-based care to enhance any treatment.* Cognitive and behavioral practice, 2015. **22**(1): p. 49-59.
35. Bickman, L., et al., *Effects of routine feedback to clinicians on mental health outcomes of youths: Results of a randomized trial.* Psychiatric Services, 2011. **62**(12): p. 1423-1429.
36. Shimokawa, K., M.J. Lambert, and D.W. Smart, *Enhancing treatment outcome of patients at risk of treatment failure: meta-analytic and mega-analytic review of a psychotherapy quality assurance system.* Journal of consulting and clinical psychology, 2010. **78**(3): p. 298.
37. Tam, H. and K. Ronan, *The application of a feedback-informed approach in psychological service with youth: Systematic review and meta-analysis.* Clinical psychology review, 2017. **55**: p. 41-55.

For peer review only

38. Bauer, A.M., et al., *Comparing Approaches to Mobile Depression Assessment for Measurement-Based Care: Prospective Study.* Journal of Medical Internet Research, 2018. **20**(6): p. e10001.
39. Woltmann, E., et al., *Comparative effectiveness of collaborative chronic care models for mental health conditions across primary, specialty, and behavioral health care settings: systematic review and meta-analysis.* Am J Psychiatry, 2012. **169**(8): p. 790-804.
40. LaMonica, H.M., et al., *Informing the Future of Integrated Digital and Clinical Mental Health Care: Synthesis of the Outcomes From Project Synergy.* JMIR Mental Health, 2022. **9**(3): p. e33060.
41. Hickie, I.B., *Implementing 21st century "end-to-end" and technology-enhanced care for young people.* World Psychiatry, 2022. **21**(1): p. 79.
42. Hickie, I.B., et al., *Right care, first time: a highly personalised and measurement-based care model to manage youth mental health.* Med J Aust, 2019. **211 Suppl 9**(9): p. S3-S46.
43. Goldman, H.H., A.E. Skodol, and T.R. Lave, *Revising axis V for DSM-IV: a review of measures of social functioning.* Am J Psychiatry, 1992. **149**(9): p. 1148-1156.
44. Iorfino, F., et al., *Delineating the trajectories of social and occupational functioning of young people attending early intervention mental health services in Australia: a longitudinal study.* BMJ Open, 2018. **8**(3): p. e020678.
45. Cross, S.P., D.F. Hermens, and I.B. Hickie, *Treatment patterns and short-term outcomes in an early intervention youth mental health service.* Early Interv Psychiatry, 2016. **10**(1): p. 88-97.
46. Cross, S.P., et al., *Variability in Clinical Outcomes for Youths Treated for Subthreshold Severe Mental Disorders at an Early Intervention Service.* Psychiatr Serv, 2018. **69**(5): p. 555-561.
47. Iorfino, F., et al., *A Digital Platform Designed for Youth Mental Health Services to Deliver Personalized and Measurement-Based Care.* Frontiers in Psychiatry, 2019. **10**.
48. Hickie, I.B., et al., *Right care, first time: a highly personalised and measurement-based care model to manage youth mental health.* Med J Aust, 2019. **211 Suppl 9**: p. S3-S46.
49. Fowler, D., et al., *Social recovery therapy in combination with early intervention services for enhancement of social recovery in patients with first-episode psychosis (SUPEREDEN3): a single-blind, randomised controlled trial.* Lancet Psychiatry, 2018. **5**(1): p. 41-50.
50. Scaini, S., et al., *A comprehensive meta-analysis of cognitive-behavioral interventions for social anxiety disorder in children and adolescents.* Journal of Anxiety Disorders, 2016. **42**: p. 105-112.
51. Carpenter, J.K., et al., *Cognitive behavioral therapy for anxiety and related disorders: A meta-analysis of randomized placebo-controlled trials.* Depress Anxiety, 2018. **35**(6): p. 502-514.
52. Robillard, R., et al., *Parallel Changes in Mood and Melatonin Rhythm Following an Adjunctive Multimodal Chronobiological Intervention With Agomelatine in People With Depression: A Proof of Concept Open Label Study.* Front Psychiatry, 2018. **9**: p. 624.
53. Wu, J.Q., et al., *Cognitive Behavioral Therapy for Insomnia Comorbid With Psychiatric and Medical Conditions: A Meta-analysis.* JAMA Intern Med, 2015. **175**(9): p. 1461-72.
54. Cunningham, J.E.A., J.A. Stamp, and C.M. Shapiro, *Sleep and major depressive disorder: a review of non-pharmacological chronotherapeutic treatments for unipolar depression.* Sleep Med, 2019.

For peer review only

55. Kopelowicz, A., R.P. Liberman, and R. Zarate, *Recent advances in social skills training for schizophrenia.* Schizophr Bull, 2006. **32 Suppl 1**(Suppl 1): p. S12-23.
56. Eisner, L., et al., *Dialectical Behavior Therapy Group Skills Training for Bipolar Disorder.* Behav Ther, 2017. **48**(4): p. 557-566.
57. Ritschel, L.A., N.E. Lim, and L.M. Stewart, *Transdiagnostic Applications of DBT for Adolescents and Adults.* Am J Psychother, 2015. **69**(2): p. 111-28.
58. McGorry, P. and I.B. Hickie, *Clinical Staging in Psychiatry: Making Diagnosis Work for Research and Treatment*. 2019, Cambridge: Cambridge University Press.
59. Lee, Y.Y., et al., *Economic evaluation of the Target-D platform to match depression management to severity prognosis in primary care: A within-trial cost-utility analysis.* PloS one, 2022. **17**(5): p. e0268948.
60. Chatterton, M.L., et al., *Economic evaluation of stepped care for the management of childhood anxiety disorders: Results from a randomised trial.* Australian & New Zealand Journal of Psychiatry, 2019. **53**(7): p. 673-682.
61. Keetharuth, A.D., et al., *Recovering Quality of Life (ReQoL): a new generic self-reported outcome measure for use with people experiencing mental health difficulties.* The British Journal of Psychiatry, 2018. **212**(1): p. 42-49.
62. Keetharuth, A.D., et al., *Estimating a preference-based index for mental health from the recovering quality of life measure: valuation of recovering quality of life utility index.* Value in Health, 2021. **24**(2): p. 281-290.
63. Reeve, R., *Confidence interval of difference of proportions in logistic regression in presence of covariates.* Statistical methods in medical research, 2018. **27**(2): p. 451-465.
64. Harris, P.A., et al., *Research electronic data capture (REDCap)—a metadata-driven methodology and workflow process for providing translational research informatics support.* Journal of biomedical informatics, 2009. **42**(2): p. 377-381.

For peer review only

Figure Legends

Figure 1: Study design and service subgroups. An overview of how the trial design gives rise to distinct groups within a single participating service. There are two implementation phases and three research arms associated with this trial which result in four distinct groups for each service based on a young person's exposure and trial participation status. Group 1 is used to establish baseline outcome statistics for the service prior to the trial commencing. Groups 2, 3 and 4 differ based on the trial status which will determine what treatments they receive. The primary outcome analysis for the RCT will be between groups 3 and 4. Routine outcome evaluation data collection is ongoing from the first phase of the trial whereby all groups will be followed up using the same processes and practices. BAU= Business as usual.

Figure 2: Study flow diagram (CONSORT style)

Figure 3. An example dashboard of results from the Innowell Platform

For peer review only

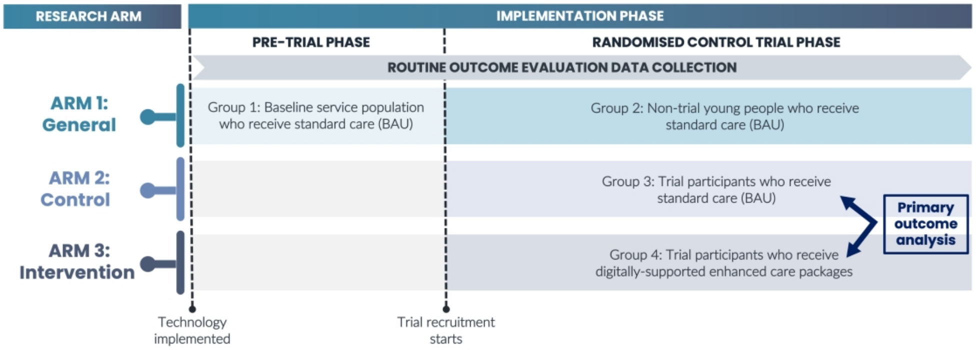

Figure 1: An overview of how the trial design gives rise to distinct groups within a single participating service. There are two implementation phases and three research arms associated with this trial which result in four distinct groups for each service based on a young person's exposure and trial participation status. Group 1 is used to establish baseline outcome statistics for the service prior to the trial commencing. Groups 2, 3 and 4 differ based on the trial status which will determine what treatments they receive. The primary outcome analysis for the RCT will be between groups 3 and 4. Routine outcome evaluation data collection is ongoing from the first phase of the trial whereby all groups will be followed up using the same processes and practices. BAU= Business as usual.

296x108mm (300 x 300 DPI)

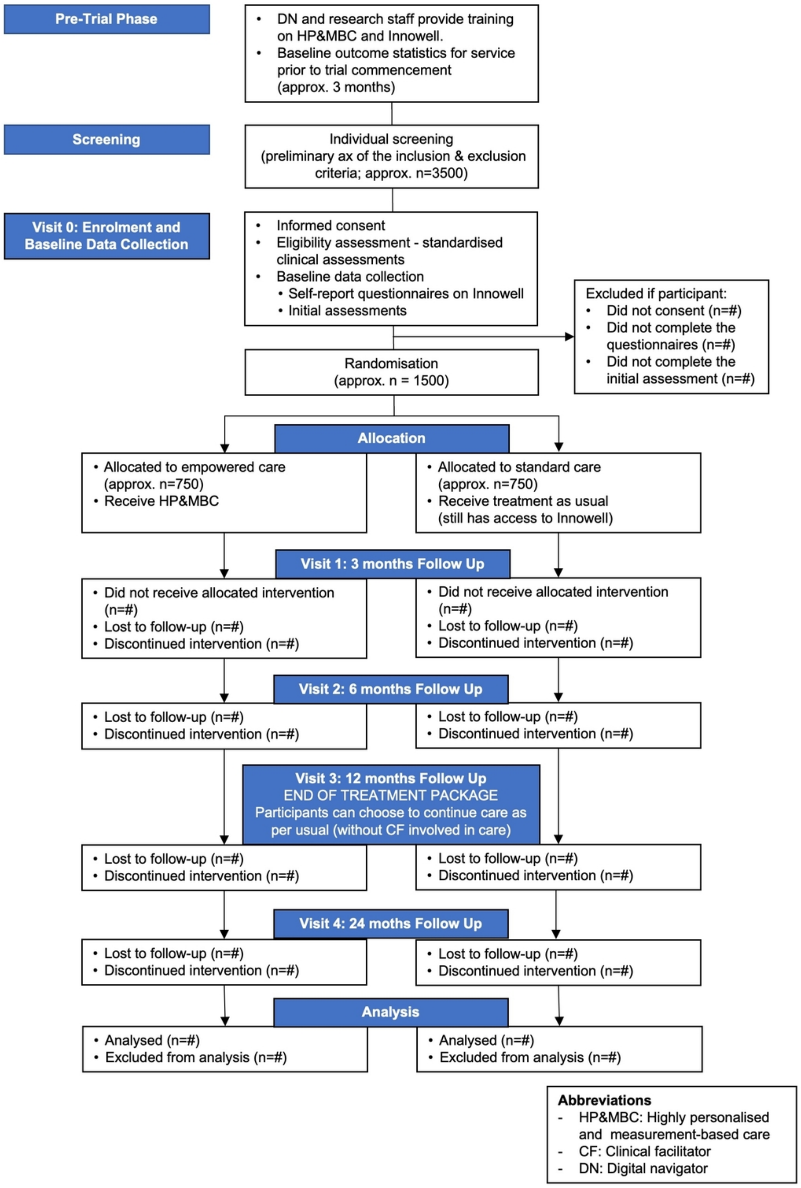

Figure 2: Study flow diagram (CONSORT style)

87x129mm (300 x 300 DPI)

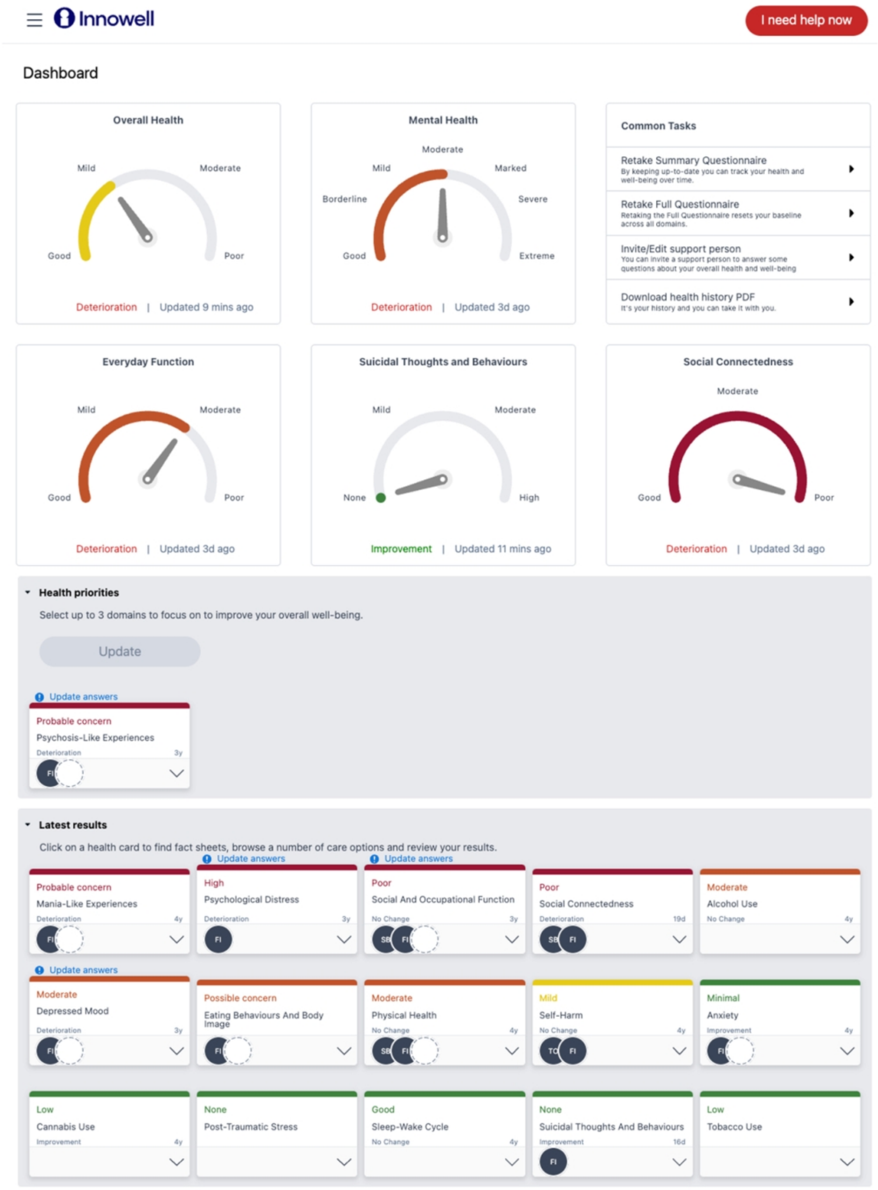

Figure 3. An example dashboard of results from the Innowell Platform

108x147mm (300 x 300 DPI)

BMJ Open

The EMPOWERED trial: Protocol for a randomised control trial of digitally supported, highly personalised and measurement-based care to improve functional outcomes in young people with mood disorders

Journal:	*BMJ Open*
Manuscript ID	bmjopen-2023-072082.R1
Article Type:	Protocol
Date Submitted by the Author:	19-Jul-2023

Complete List of Authors:	Hickie, Ian; The University of Sydney Brain and Mind Centre, Brain and Mind<br>Iorfino, Frank; The University of Sydney Brain and Mind Centre, Brain and Mind Centre<br>Rohleder, Cathrin; The University of Sydney Brain and Mind Centre<br>Song, Yun; The University of Sydney, Brain and Mind Centre<br>Nichles, Alissa; The University of Sydney, Brain and Mind Centre<br>Zmicerevska, Natalia; The University of Sydney, Brain and Mind Centre<br>Capon, William; The University of Sydney Brain and Mind Centre, Brain and Mind Centre<br>Guastella, Adam; Brain & Mind Research Institute, Clinical Research Unit<br>Leweke, F. Markus; The University of Sydney, Brain and Mind Centre; Ruprecht Karls University Heidelberg Faculty of Medicine Mannheim, Psychiatry and Psychotherapy, Central Institute of Mental Health<br>Scott, Jan; Newcastle University, Institute of Neuroscience<br>McGorry, Patrick; Oryegn Research Centre, University of Melbourne Australia<br>Mihalopoulos, Cathrine; Monash University, School of Public Health and Preventive Medicine<br>Killackey, Eoin; Orygen, The National Centre of Excellence in Youth Mental Health; University of Melbourne, Centre for Youth Mental Health<br>Chong, Min Kyung; The University of Sydney Brain and Mind Centre<br>McKenna, Sarah; The University of Sydney Brain and Mind Centre<br>Aji, Melissa; The University of Sydney Brain and Mind Centre<br>Gorban, Carla; The University of Sydney Brain and Mind Centre<br>Crouse, Jacob; The University of Sydney, Brain and Mind Centre<br>Koethe, Dagmar; The University of Sydney, Brain and Mind Centre<br>Battisti, Robert; Mind Plasticity<br>Hamilton, Blake; The University of Sydney, Brain and Mind Centre; headspace Camperdown<br>Lo, Alice; Mind Plasticity<br>Hackett, Maree; George Institute for Global Health, Faculty of Medicine, University of New South Wales; University of Central Lancashire, Faculty of Health and Wellbeing<br>Hermens, Daniel; Sunshine Coast Mind and Neuroscience Thompson Institute,<br>Consortia, Mind Plasticity; Mind Plasticity<br>Consortia, headspace Camperdown; headspace Camperdown

	Scott, Elizabeth; Brain & Mind Research Institute,
**Primary Subject Heading**:	Mental health
Secondary Subject Heading:	Mental health
Keywords:	MENTAL HEALTH, PSYCHIATRY, Clinical trials < THERAPEUTICS

SCHOLARONE™ Manuscripts

The EMPOWERED trial: Protocol for a randomised control trial of digitally supported, highly personalised and measurement-based care to improve functional outcomes in young people with mood disorders

Ian B Hickie[1], Frank Iorfino[1*], Cathrin Rohleder[1], Yun Ju Christine Song[1], Alissa Nichles[1], Natalia Zmicerevska[1], William Capon[1], Adam J Guastella[1], F Markus Leweke[1,2], Jan Scott[3], Patrick McGorry[4,10], Cathrine Mihalopoulos[5], Eoin Killackey[4, 10], Min Kyung Chong[1], Sarah Mckenna[1], Melissa Aji[1], Carla Gorban[1], Jacob Crouse[1], Dagmar Koethe[1], Robert Battisti[6], Blake Hamilton[1,7], Alice Lo[6], Maree L Hackett[8], Daniel F Hermens[9], Mind Plasticity Consortia[6], headspace Camperdown Consortia[7] & Elizabeth M Scott[1]

[1] Brain and Mind Centre, The University of Sydney, Sydney, New South Wales, Australia
[2] Dept of Psychiatry and Psychotherapy, Central Institute of Mental Health, Medical Faculty Mannheim, Heidelberg University, Mannheim, Germany
[3] Newcastle University, Newcastle upon Tyne, England, United Kingdom
[4] Centre for Youth Mental Health, University of Melbourne, Melbourne, Victoria, Australia
[5] School of Public Health and Preventive Medicine, Monash University, Victoria, Australia
[6] Mind Plasticity, Sydney, New South Wales, Australia
[7] headspace Camperdown, Sydney, New South Wales, Australia
[8] The George Institute for Global Health, Sydney, New South Wales, Australia
[9] Thompson Institute, University of the Sunshine Coast, Birtinya, Queensland, Australia.
[10] Orygen, Melbourne, Australia.

Correspondence to:
Frank Iorfino, 94 Mallet Street Camperdown NSW 2050, frank.iorfino@sydney.edu.au

Sources of support: This study is supported by a National Health and Medical Research Council (NHMRC) Clinical Trials and Cohort Studies grant (grant number: 2001568).

Word Count: 4527 (including Textbox 1, excluding Tables and Table legends)

Number of figures and tables: 5 (3 figures, 2 tables)

For peer review only - http://bmjopen.bmj.com/site/about/guidelines.xhtml

Abstract (300 words)

Objectives

Many adolescents and young adults with emerging mood disorders do not achieve substantial improvements in education, employment, or social function after receiving standard youth mental health care. We have developed a new model of care referred to as 'highly personalised and measurement-based care' (HP&MBC). HP&MBC involves repeated assessment of multidimensional domains of morbidity to enable continuous and personalised clinical decision-making. Although measurement-based care is common in medical disease management, it is not standard practice in mental health. This clinical effectiveness trial tests whether HP&MBC, supported by continuous digital feedback, delivers better functional improvements than standard care and digital support.

Method and analysis

This controlled implementation trial is a PROBE study (Prospective Randomized Open, Blinded End-point) that comprises a multi-site 24-month, assessor-blinded, follow-up study of 1500 individuals aged 15-25 years who present for mental health treatment. Eligible participants will be individually randomized (1:1) to 12 months of HP&MBC or standardised clinical care. The primary outcome measure is social and occupational functioning 12 months after trial entry, assessed by the Social and Occupational Functioning Assessment Scale (SOFAS). Clinical and social outcomes for all participants will be monitored for a further 12 months after cessation of active care.

Ethics and dissemination

This clinical trial has been reviewed and approved by the Human Research Ethics Committee of the Sydney Local Health District (HREC Approval Number: X22-0042 & 2022/ETH0072, Protocol ID: BMC-YMH-003-2018, protocol version: V.3, 03/08/2022). Research findings will be disseminated through peer-reviewed journals and presentations at scientific conferences and to user and advocacy groups. Participant data will be de-identified.

Trial registration number ACTRN12622000882729

For peer review only

For peer review only - http://bmjopen.bmj.com/site/about/guidelines.xhtml

Strengths and limitations of this study

Up to five short bullet points, no longer than one sentence each, that relate specifically to the methods.

1. To our knowledge, this is the first large scale effectiveness trial that tests whether early intervention and secondary prevention deliver substantive improvements in functional outcomes for young people with major mood disorders.
2. The trial sample will be large, and the use of minimal eligibility criteria maximises the generalisability of these findings to other youth mental health settings
3. The trial introduces new service roles (i.e., 'digital navigator', 'clinical facilitator') to help clinicians and clients to access the optimal package of interventions.
4. Standard care packages are delivered in the same setting and by the same health professional as the intervention group. So cross-over effects may attenuate between groups differences.
5. The availability and access to specific interventions needed to deliver enhanced care to those in the intervention group may be variable across different trial sites.

For peer review only

Introduction

There has been increasing recognition of the premature death and persistent disability attributable to the major mental disorders[1, 2]. The largest proportion of this excessive morbidity is attributable to mood disorders, reflecting their early age of onset, high population prevalence, chronicity and comorbidity [3]. While significant investments have been made in youth mental health services internationally, there is a lack of substantive evidence for which models of care are optimal for improving illness outcomes.

Mood disorders place young people at risk of prolonged socioeconomic difficulties, even when their mental ill-health subsides[4, 5]. Our work has identified that up to two-thirds of young people in youth mental health services experience poor longer-term functional outcomes [6, 7]. Current evidence suggests that youth mental health services primarily benefit those in the earlier stages of illness and that while brief psychological interventions are effective for reducing psychological distress, they only marginally improve functioning[8]. Further, current models of care do not appear to be well suited to those with comorbidities mixed or attenuated symptomatology, or social and occupational complexity. Most treatment plans are focused narrowly on limited treatment choices or 'steps' for discrete disorders. They are based on average population effects or clinical experience [9-17], and are often inaccurate and inconsistent.

The differentiation of young people with 'reasonable/good' from 'impaired/poor' functioning at presentation is a key factor to be considered (alongside other clinical variables) to determine the need for highly personalised care with the appropriate type, intensity, sequence, and duration of multidisciplinary interventions. This approach aligns with optimal models of mental health care and should be a key component of youth mental health service provision[18]. Yet, the evidence-base for health service models that guide personalised interventions for young people with mood disorders is sparse[19-22]. Furthermore, it is not standard practice to use measurement-based care (MBC) for the monitoring of symptoms and functioning to drive continuous and personalised clinical decision making[23-27]. Highly personalised and measurement-based care, which entails routine assessment of multidimensional outcomes and regular monitoring of an individual's response to treatment, is a core component of the chronic care model and supports better-informed clinical decisions[28-34]. These decisions may include the adjustment of treatment type and intensity. Despite good evidence for its effectiveness and its customary use in physical disease management[31, 32], it remains largely absent from youth mental health care[9, 35].

Objectives of the study

The primary objective of this large-scale clinical effectiveness trial is to assess the effectiveness of 12 months of intensive, personally tailored, assertive care (the digitally supported HP&MBC package), compared with digitally-supported standard clinical care. We will test whether the HP&MBC package results in a greater improvement of social and occupational functioning

For peer review only - http://bmjopen.bmj.com/site/about/guidelines.xhtml

compared to standard clinical care. The secondary objective is to assess the mental health status of all participants 12 months after the enhanced and standard care interventions.

We hypothesise that while the standard care packages will be an improved offering (through greater standardisation of assessment and access to digital technology), the HP&MBC treatment packages will be superior by implementing continuous and proactive monitoring and care coordination using digital technologies and providing extensive feedback to the clinical service, the treating clinician, the young person and their family or carer[36].

Methods and analysis

Study design and setting

This large-scale, prospective study aims to enrol 1500 mental health treatment seeking young people with mood disorders. The trial was designed with the aid of young people with lived experience of mental illness and is a PROBE study (Prospective Randomized Open, Blinded End-point). It comprises a 24-month (12 months active treatment, 12 months additional follow-up), multi-site, two-arm (HP&MBC care package vs standardised clinical care), randomised (1:1), blinded outcome assessor, controlled implementation trial. The trial will be conducted at the Brain and Mind Centre (The University of Sydney, Australia) and affiliated youth centres that focus on treating young people with mental illness. As noted below, prior to commencing the RCT, there will be a pre-trial phase (Figure 1).

<u>Pre-trial phase</u>

The study includes a pre-trial phase to allow the digital technology platform to be introduced to the clinical teams and integrated into the service procedures. This period will be used to work through any implementation issues prior to commencing the RCT. Also, it permits collection of pre-trial data from each clinical service, including an audit of outcomes in routine clinical practice (e.g., rates of improvement or deterioration in social and occupational function in non-trial clinical cohorts).

<u>RCT and follow up phase (~24 months)</u>

After the pre-trial phase, the RCT phase of the study commenced in early 2023, with the first participant enrolled on 1/03/2023. Participation in the trial will be 24 months following enrolment (baseline assessment), including 12 months of active treatment and 12 months additional follow-up. Five independent assessments will be conducted: at baseline, three months, six months, 12 months, and 24 months. We anticipate recruitment and randomization of 100% of the target sample size by the end of 2025, and we estimate data collection to be completed by late 2027.

Patient and public involvement

For peer review only - http://bmjopen.bmj.com/site/about/guidelines.xhtml

Young people with lived experience of mental illness were invited to participate in the study design through consultation with the Brain and Mind Centre Lived Experience Working Group. The working group consists of culturally and linguistically diverse young people aged 16-30 years old. The principles underpinning the trial, and the name for this trial 'EMPOWERED', were identified by young people with lived experience (textbox 1).

Study Population

The study focuses on young people seeking help for psychological distress and presenting with early stage mood syndromes, characterised not only by the mix of anxiety or depressive symptoms, and their impact on function, but also according to stage of illness criteria (Stage 1a – non-specific anxiety and depressive syndromes), attenuated syndromes (Stage 1b) or first full-threshold, major and discrete mood syndrome (Stage 2)[18]. Recruitment is also based on the presentation to care and existing functional impairment. This approach is consistent with the National Institute of Mental Health recommendations for conducting more integrative clinical research[14]. Approximately 10,000 individuals aged 15-25 years present to the Brain and Mind Centre and affiliated youth centres per year. We expect that 3000 individuals will meet the inclusion criteria and that about 50% of the eligible individuals will consent. In total, 1500 young people will be included (750 allocated to the active 12-month care package and 750 allocated to the standardised care package).

Textbox 1. The EMPOWERED trial principles

1. **E**ducate – To educate young people, and their families and carers, on the potential usefulness of technology, and how routine monitoring can give them a greater say in their care journey.
2. **M**easurement-based – To improve continuous and real-time measurement of young people's symptoms and functioning, and longer-term outcomes, so that they can receive more effective care.
3. **P**ersonalised – Ensuring that treatment is personalised, so that the complexity of young people's needs are recognised, documented, acted on and preserved over the care journey.
4. **O**penness – Improving open communication between young people, their families and carers, and clinicians by making everyone more informed about progress in care.
5. **W**ork collaboratively – Helping clinicians and young people to work collaboratively to create and respond to treatment goals by facilitating treatment monitoring, emphasizing functional recovery, and allowing young people to focus on assessment domains that matter most to them.
6. **E**ngage – Increasing young people's engagement in care planning, by putting information about their mental health into their own hands.
7. **R**ecovery – Earlier recovery through improved clinical and functional assessment, and actively monitoring social, education and employment outcomes, to ensure that young people receive earlier and more personalised care.
8. **E**nhanced **D**igitally – Leverage the advanced capabilities of digital technologies to facilitate the assessment, monitoring and management of mental health problems, and support shared and informed decision making.

Inclusion and exclusion criteria

For peer review only - http://bmjopen.bmj.com/site/about/guidelines.xhtml

Participation in this study will be offered to adolescents and young adults aged 15- 25-years seeking help for psychological distress and classified as Stage 1a, 1b or 2. The participants must have an initial Social and Occupational Functioning Assessment Score (SOFAS) of ≤ 70[37], indicating impaired social and occupational functioning. Young people with a lifetime diagnosis of a full-threshold psychotic or bipolar I disorder, or alcohol or other substance dependence, will be excluded. Additional exclusion criteria include acute suicidal or aggressive behaviour requiring alternative care or a depressive syndrome secondary to a primary medical condition. Young people who have a clinically evident intellectual disability (IQ<70 as per medical history review) will be excluded due to the likely difficulty in completing the assessments.

Study course and procedures

The clinical trial comprises 12-months active treatment and 12-months follow-up phase, i.e., each subject will be followed for two years, whereby five blinded independent assessment visits will take place (Figure 2).

Individuals referred to the trial will be contacted by a research team member who will provide information about the study and conduct a preliminary assessment of the inclusion and exclusion criteria. Potential participants interested in taking part in the study will then be provided with a copy of the participant information statement (Supplemental Material 1), and an appointment will be scheduled for an enrolment visit. During the enrolment/baseline visit (Visit 0), the study will be explained in lay terms and any questions will be answered. Following informed consent, participants will be given relevant assessments to confirm that they meet the inclusion criteria.

Participants who meet the inclusion criteria will be randomised to one of the two treatment arms using a 1:1 individual person randomisation algorithm (using REDCap) taking age, gender and treatment centre as stratification factors into account. The care packages will be delivered within the first 12 months of the study by clinicians operating within each service. During this study phase, three study visits will take place: (i) Visit 1 (3 months after trial entry); (ii) Visit 2 (6 months after trial entry); and (iii) Visit 3 (12 months after trial entry). A follow-up visit (Visit 4) will be conducted 12 months after completion of the care packages (Figure 2). All follow up assessments will be carried out by blinded-independent assessors from the research team.

Care Packages

To coordinate ongoing clinical care and functional recovery, real-time data feedback will be provided in both treatment arms using health information technology. As demonstrated in our longitudinal studies[7, 8, 38], those with attenuated syndromes often receive only brief interventions (six or fewer sessions of psychological support) and exit services with residual high levels of impairment. There is a fundamental mismatch between the time course of impairment (typically well-established by the time the young person presents to clinical care) and the brief clinical interventions provided by early intervention services. Therefore, we now

For peer review only - http://bmjopen.bmj.com/site/about/guidelines.xhtml

use personalised technologies to tailor, plan, and track the relationships between clinical care delivery (and sequencing) and functional recovery strategies. The real-time data feedback will support optimal combinations of indirect and direct intervention strategies until the young person achieves: (i) syndromal remission and risk reduction and (ii) social and occupational recovery. This real-time data feedback will be supported by the Innowell Platform[39].

The digitally supported HP&MBC care package represents an intensive, personalised and assertive treatment package. It builds on the usual processes provided by the services at each centre, including systematic assessment and allocation of clinical care within multidisciplinary team environments. The HP&MBC care package uses two key streams, namely (a) the therapeutic power of active and continuous feedback with regards to illness type, course, response to interventions and social and economic impact of care; and (b) the capacity of new assessment and monitoring techniques to tailor treatment options – with the standardisation of those stepped-care options into an on-going and proactive shared care plan.

The HP&MBC enhanced care package includes:

(i) Initial digital assessment covering the domains of symptoms, social and occupational functioning, self-harm and suicidal thoughts and behaviours (STBs), physical health and alcohol and other substance misuse;

(ii) Feedback of the initial 'dashboard' of results to the user of care and family members, clinical services and the principal treating clinician (Figure 3);

(iii) Continuous outcome monitoring and feedback – Regular review of 'dashboard' to the user of care and family members, clinical services, and principal treating clinician (monthly for first 12-months, may vary based on individual needs);

(iv) More detailed online, clinical, neuropsychological and lab-based testing as recommended by digital or clinical protocols, including use of specific individual monitoring devices (e.g., wearable activity monitors, mood monitors) to inform broad diagnostic categorisation and then assign a more specific series of highly personalised treatment options;

(v) Determination of indicative sub-type of depressive syndrome by incorporation of clinical factors and life course, to link to specific intervention strategies;

(vi) Utilisation of online shared care planning by the user of care and family members, clinical services, and principal treating clinician;

Active and continuous feedback will guide evidence-based decision making related to treatment plans as it supports the choice of optimal combinations of interventions. The measurement-based feedback will help detect unmet needs, increase the likelihood that clinicians identify young persons who are non-responsive to treatment, and facilitate the process to adjust the plan of care to improve young person outcomes.

For peer review only - http://bmjopen.bmj.com/site/about/guidelines.xhtml

The standard care package builds on the usual service systems (largely Medicare-funded psychological care), including systematic assessment and allocation of clinical care within multidisciplinary team environments.

The standardised care package includes:

(i) Initial digital assessment covering the domains of symptoms, social and occupational function, self-harm, and STBs, physical health and alcohol and other substance misuse;

(ii) Feedback of the initial 'dashboard' of results to the user and treating clinician at baseline;

(iii) Provision of standard multidisciplinary care options and ongoing access to other relevant psychological and pharmacological options;

In this study, the following targeted therapies (over and above standard psychological care), which have been shown in various studies to have beneficial effects[18, 40-48], are of particular relevance:

(i) *Social and Functional Recovery Therapies:* Interventions that target social recovery include direct support to return to work, re-engaging in education or training, and social skills training to reduce isolation and improve relationships with peers and family. Key components of these interventions include setting meaningful recovery goals, establishing the external resources to support recovery, and using outreach graded behavioural experiments to re-establish functioning.

(ii) *Circadian Interventions:* Pharmacological (e.g., agomelatine, brexpiprazole), physical (e.g., light therapy) or behavioural interventions (e.g., sleep-wake rescheduling) that target dysregulation of sleep-wake behaviours and biological circadian rhythms.

(iii) *Cognitive-Behaviour Therapies (CBT) and Social Therapies Groups:* CBT teaches the individual to link their feelings, thoughts, and patterns of behaviours to reduce psychological distress. A greater focus on social cognition training may be needed for those with social cognitive impairment.

(iv) *Dialectic Behaviour Therapy (DBT):* DBT is a modified version of CBT designed to treat symptoms often associated with emotional dysregulation and poor distress tolerance such as self-harm, suicidal behaviour, and substance use. The emphasis is on moving away from harmful coping behaviours and incorporates mindfulness, distress tolerance, emotional regulation, and interpersonal effectiveness strategies.

(v) *Healthy lifestyle and cardiometabolic health targeted treatments:* The World Health Organisation guidelines recommend that lifestyle behavioural interventions be considered the first-line treatments for managing physical health (including cardiometabolic health) for those with severe mental illness. Psychoeducational interventions focusing on healthy lifestyle habits including diet, physical activity

For peer review only - http://bmjopen.bmj.com/site/about/guidelines.xhtml

and sleep practices have been shown to ameliorate both the physical and mental health concerns of young people with psychiatric disorders.

Whilst these therapies will be available to participants in both treatment arms, those in the HP&MBC treatment group will be actively referred to the specific optimal treatment program/s based on the outcomes of the continuous assessment data that will be made available to the participant and their treating clinician. In addition to the targeted therapies mentioned above, additional relevant therapies may be introduced over the course of the study as the clinical needs of participants become apparent.

Service roles

Two additional service roles will be employed for the trial (Table 1). The first is a 'clinical facilitator' who is an independent clinician focused on ensuring optimal uptake of the HP&MBC by the treating clinicians. This will be achieved by working collaboratively with clinicians with the aim of reducing the additional tasks associated with enhanced and rapid communication, tracking, and interpreting and actioning feedback. The second role is a 'digital navigator' who will operate for participants across both arms of the trial. The primary focus of the role is to provide peer support for young people to motivate them to provide outcome data; regularly remind them of the purpose of collecting data and how it can improve their treatment journey; help the young person, carer and clinician to address technical issues; and provide guidance about useful e-tools to be used in treatment.

For peer review only

Table 1. Role description of the facilitator team.

Description of Roles	Tasks	Examples
Clinical Facilitator<br>*Only available for participants intervention arm*<br><br>The purpose of this role is to facilitate the use of the HP&MBC by clinicians. This is achieved by working collaboratively with clinicians with the aim of reducing burden associated with communication, tracking, and interpreting and actioning feedback. The main responsibilities of this role include:<br>a) Assisting clinicians to review and aid identification of any domains of concern (e.g. increased risk or decreased social support);<br>b) Providing logistical support in making referrals for clients<br><br>This role does not have any clinical responsibility towards clients as this is a support role.	• Promote and assist with the use of routine client feedback to inform personalised treatment options<br>• Reduce time burden for clinicians by monitoring client progress using technology and alerting clinicians if significant deteriorations/risk arise<br>• Performing administrative tasks to facilitate referrals, and identify where appropriate treatment options recommended by the youth model<br>• Regularly assess with clinicians how client feedback has been used in sessions to inform treatment<br>• Develop a good understanding of referral options in the relevant area including community organisations, schools, public health services, online services and apps, etc<br>• Assist with identifying appropriate care options and help with the logistics of the organisation of clinical care.	**SOFAS deterioration**<br>Clinical facilitator (CF) notes that patient X's SOFAS has deteriorated ten points since their last report one month ago. CF communicates with X's psychiatrist, using their preferred communication method, letting them know that there has been a deterioration. Psychiatrist notifies CF that they have commenced a new course of treatment at their last appointment two weeks ago and will continue monitoring their symptoms. CF also communicates with psychologist to let them know about deterioration and notes that psychiatrist has changed medication recently. Psychologist notes that client X has recently begun exposure exercises in their weekly therapy sessions that they are finding highly distressing.<br><br>One month later, the client reports further deterioration to SOFAS and that they have experienced an increase in passive suicidal ideation. CF communicates this to the psychiatrist and psychologist. Psychiatrist requests DBT and CF facilitates meeting between psychologist and psychiatrist to discuss options.<br><br>CF also contacts three local community and public health services that offer DBT programs and finds that Cremorne Health Centre has a spot available for client X. CF passes this information to psychiatrist to make referral.
Digital Navigator<br>*Available to participants in both arms of the trial*	• Troubleshoot any issues related to technology for clients, caregivers and clinicians<br>• Remind clients to complete	**Enrolment of a new participant**<br>Client X newly joined the trial. The DN will organise a brief meeting with the client to introduce

For peer review only - http://bmjopen.bmj.com/site/about/guidelines.xhtml

The primary focus of the role is to:<br>a) provide peer support for clients to motivate them to provide outcome data - regularly remind the clients of the purpose of collecting data and how it can improve their treatment journey;<br>b) help client, carer and clinician to address other technical issues;<br>c) provide guidance about useful etools (online resources and apps) to be used in treatment;	Innowell questionnaires. Routinely follow up with clients, through their preferred method (e.g. text, email or face to face) to ensure regular data collection<br>• Research evidence-based etools that clinicians can confidently use as part of treatment	Innowell and to educate them on the purpose of using the platform and its potential benefits.<br><br>After 1 month, DN follows up with client X to collect feedback about their experience of Innowell and whether regular reporting about their symptoms has been used by clinicians to inform treatment.<br><br>Client X states that they liked how their functional scores were discussed during the session but wished that their physical status was addressed. DN relays the feedback and suggest an app that can monitor client physical status to CF. CF alerts clinicians about client X's physical scores and promotes active response.

Assessments

A series of standardised clinical assessments will be conducted at the enrolment visit (Visit 0) to assess inclusion and exclusion criteria (Table 2), including:

1. Structured Clinical Interview for DSM-5 to assess the presence of mental health and substance use disorders.
2. A framework for clinical staging[18, 49] will be applied to assess illness severity and differentiate those in the earliest phases with non-specific clinical presentations (stages 1a 'seeking help') from those at greater-risk with more specific, sub-threshold presentations (stage 1b 'attenuated syndromes') and those who have reached a threshold for a progressive or recurrent disorder meeting diagnostic criteria (stage 2, 3, or 4).
3. Social and Occupational Functioning Assessment Scale (SOFAS) to record the clinician's judgement of overall social and occupational function.
4. A mental risk assessment to assess acute suicidal behaviour.

As summarised in Table 2, individuals who fulfil all inclusion and exclusion criteria will undergo additional clinician/researcher-administered baseline assessments evaluating depressive symptomatology, personal social performance, and self-report questionnaires will be provided to collect information regarding the quality of life, self-harm, suicidal thoughts and behaviours, alcohol and substance use, and physical health. Furthermore, blood will be collected to assess metabolic, inflammatory and standard blood markers.

For peer review only - http://bmjopen.bmj.com/site/about/guidelines.xhtml

While social and occupational functioning, illness severity, and depressive symptoms will be assessed at every subsequent visit (Visits 1-4), the structured clinical interview will only be repeated at the end of the active treatment phase (12 months after trial entry, Visit 3; and 24 months after trial entry, Visit 4). Self-report questionnaires will be provided at each visit during the active treatment phase (visits 1-3). Blood samples will be collected at baseline, 6 months, 12 months and 24 months after trial entry (i.e., at visits 0, 2, 3 and 4) to monitor changes in metabolic, inflammatory and standard blood markers. Blood test results for young people in the intervention arm will be immediately relayed to treating clinicians, to provide more data to determine the appropriate treatment for the participant. For example, non-specific immunosuppressive therapies or innovative immune therapies could be the optimal treatment approach for young people with atypical major mood or psychotic disorders.

Resource use that will also be used to estimate costs will be measured using two main procedures:

1. Participants will be asked for access to administrative data sets including the Medicare Benefits Schedule (MBS) and Pharmaceutical Benefits Schedule (PBS) data for the duration of the study.

2. The resource use questionnaire, used in multiple mental health economic evaluations, which captures the broad range of health and welfare services used by participants and is complementary to any administrative data also included in the evaluation [50, 51].

Micro-costing techniques will be used to assess the costs of the intervention. Standardised economic evaluation techniques including incremental analysis of mean differences using generalised linear models, and bootstrapping to determine confidence intervals will also be used. Lifetime and population cost-effectiveness will be also determined using economic modelling techniques.

Table 2: Overview of research assessments. Abbreviations: AUDIT-C, Alcohol Use Disorders Identification Test – Consumption; BMI, Body Mass Index; B-NSSI-AT, Brief Non-suicidal Self-injury Assessment Tool; CAARMS (7.3), Comprehensive Assessment of At-Risk Mental States – item 7.3; C-SSRS, Columbia-Suicide Severity Rating Scale; DSM-5, Diagnostic and Statistical Manual of Mental Disorders, 5th edition; IPAQ, International Physical Activity Questionnaire – short version; PSP, Personal and Social Performance Scale; QIDS-C, Quick Inventory of Depressive Symptomatology – Clinician-rated; ReQoL-10, Recovering Quality of Life Questionnaire (10-item version); SIDAS, Suicidal Ideation Attributes Scale; SOFAS, Social and Occupational Functioning Assessment Scale; WHO-ASSIST, World Health Organisation Alcohol, Smoking and Substance Involvement Screening Test (version 3.1).

Domain	Assessment	Administration	Time points (Months)				
			0	3	6	12	24

For peer review only - http://bmjopen.bmj.com/site/about/guidelines.xhtml

**Clinical Diagnosis**	Structured Clinical Interview to assess for DSM-5 Mental Health and Substance Use Disorders	Researcher administered	✓			✓	
**Acute suicidal and aggressive behaviour (exclusion criteria)**	CAARMS (7.3 and 5.4)	Researcher administered	✓				
**Social and occupational functioning**	SOFAS	Researcher administered	✓	✓	✓	✓	✓
**Social and occupational functioning**	PSP	Researcher administered	✓	✓	✓	✓	✓
**Depressive Symptoms**	QIDS-C	Researcher administered	✓	✓	✓	✓	✓
**Illness severity**	Clinical staging	Researcher administered	✓	✓	✓	✓	✓
**Quality of life**	ReQoL-10	Self-report	✓	✓	✓	✓	✓
**Self-harm / suicidal thoughts and behaviours**	SIDAS / adaptation of the C-SSRS / B-NSS-AT	Self-report	✓	✓	✓	✓	
**Alcohol and substance use**	AUDIT-C – Alcohol use<br>WHO-ASSIST – Alcohol and other substance use	Self-report	✓	✓	✓	✓	
**Physical health**	Height / weight / waist / BMI	Self-report	✓	✓	✓	✓	
**Physical health**	IPAQ (physical activity)	Self-report	✓	✓	✓	✓	
**Physical health**	Metabolic, inflammatory & standard clinical bloods	Researcher administered	✓		✓	✓	
**Resource Use**	Resource Use Questionnaire	Self-report	✓	✓	✓	✓	✓

Primary and secondary outcomes

Primary efficacy endpoint:

- Changes in social and occupational function from baseline to 12 months, as assessed by the SOFAS.

Key secondary endpoints:

- Change from baseline in self-harm, suicidal thoughts and behaviours (B-NSSI-AT, SIDAS, C-SSRS)
- Change from baseline in depressive symptoms (QIDS)
- Change from baseline in quality of life (ReQoL)[52, 53]
- Change from baseline in alcohol and substance use (WHO-ASSIST, AUDIT-C)
- Change from baseline in physical health (IPAQ, height, weight, waist)
- Change from baseline in metabolic, inflammatory and standard blood measures (metabolic and inflammatory markers, e.g. assessment of triglycerides, cholesterol, glucose, iron)
- Resource use as well as lifetime and population cost-effectiveness.

For peer review only

For peer review only - http://bmjopen.bmj.com/site/about/guidelines.xhtml

- Costs of the treatment packages based on detailed economic evaluation.

Sample size calculation

This trial seeks to recruit 1,500 young people, with 750 allocated to active 12-month intervention and 750 to standard clinical care. We anticipate an attrition rate of approximately 10-20% over short-term follow up (first 12 months) and up to 30% over the longer-term follow-up (at 24 months). Therefore, we would expect 1350 participants at six months follow-up (675 in each arm), 1200 participants at 12 months follow-up (600 in each arm) and 1050 participants at two years follow-up (525 in each arm). Assuming that we have at least 434 young people at the two-year follow-up time point, for the primary outcome analysis only, and conservatively assuming a small effect size difference of 0.2 in favour of those young people receiving the active intervention, α=0.05, we have 95% power. For categorical secondary analyses, a small effect size of 0.2, α=0.05, power=95%, sample size at two-year follow-up is 325 participants. There are also embedded sub-groups for secondary analyses (e.g., by baseline suicidal acts, depressive sub-type, alcohol or other substance misuse and baseline SOFAS bands). For these subgroups, assuming that we have at least 195 young people at the two-year follow-up time point, for the primary outcome analysis only, and conservatively assuming a medium effect size difference of 0.3 in favour of those young people receiving the active intervention, α=0.05, we have 95% power. For categorical secondary analyses, a medium effect size of 0.3, α=0.05, power=95%, sample size at two-year follow-up is 144 participants.

Data analysis plan

The primary outcome will be analysed using a repeated-measure linear mixed model including all available SOFAS scores measured at months 3, 6, 12 and 24. Fixed effects will include the randomised group, visit as a categorical variable and the interaction between group and visit. The baseline SOFAS score will be included as a covariate alongside sex, age and site (stratification variables). To account for correlations between repeated measures, a random patient intercept will be included. In case of convergence issues with the inclusion of the random effect, we will replace the random effect with a repeated effect assuming a compound symmetry covariance structure. This model will be used to derive the effect of the intervention at 12 months, expressed as the adjusted mean difference and its 95% confidence interval. The effect of the intervention at other timepoints will be estimated using a similar approach.

Secondary outcomes will be analysed using a similar approach. For binary outcomes, logistic regression (binomial distribution with logit link) will be used in place of linear regression. The effect of the intervention will be estimated as the odds ratio and 95% confidence interval and converted to an absolute risk difference using the Hummel and Wiseman method[54]. Given that linear mixed models use all data available and make valid inference under the assumption that data is missing at random, the primary analysis will not impute missing data; however, sensitivity analyses will be conducted to assess the robustness of the results under different

For peer review only - http://bmjopen.bmj.com/site/about/guidelines.xhtml

assumptions about the missing data mechanism. A detailed statistical analysis plan including mock tables will be developed prior to unblinding and database lock.

The economic evaluation of the HP&MBC package is critical to translate this research into practice. It will comprise both a "within-trial" design whereby the individual level costs and outcomes of the two groups (HP&MBC and Standard Care packages) will be included in the evaluation over the duration of the trial. A modelled evaluation will be undertaken to capture full costs and consequences of HP&MBC, using the results of this trial and the broader epidemiological literature to estimate likely longer term health gains, cost impacts and scale up costs at the population level. The calculation of quality-adjusted life years (QALYs) will be done, thus enabling a cost-utility analysis to be undertaken. Cost-utility analyses are useful to decision-makers as they are associated with inherent value for money connotations. Detailed costing of the HP&MBC approach along with how it has been implemented within each site will be undertaken using information from the researchers, clinical staff, and budgetary personnel.

Data management and security

All data collected for the purposes of the study will be linked to unique study ID codes and will not contain identifying information. Data collection will be conducted only by authorised members of study staff, to whom this duty has been allocated and who are named on the Human Ethics application and Governance approvals for the trial. Research data will be stored in REDCap and electronic data generated by participant outcomes will be electronically stored via the Innowell Platform.

Any publications or reports based on this study will include only pooled results from participants. Routine internal audits data files will ensure completeness of data collection. Data for which hardcopies are generated will be stored in both original hard copy and electronic form. Hardcopies will be retained so that comparison between electronic and original data is possible to ensure accuracy of data entry and resolve issues concerning spurious data in the electronic file. This data will be kept under 1) lock and key at trial site or 2) electronic file that is password protected and accessible only by research staff responsible for data entry or monitoring.

Monitoring will be done by Investigator Christine Song as she is removed from the day-to-day activities and has CRA experience. This will be at site initiation, after the first 50 patients are enrolled and then 6-monthly after, and at the close-out visit. The monitoring visits will involve a self-audit checklist, 10% source data verification, review of adverse events and serious adverse events, inclusion and exclusion criteria review, and a protocol deviation review.

Participant safety

For peer review only - http://bmjopen.bmj.com/site/about/guidelines.xhtml

Safety reporting is subject to the NHMRC's guidance on Safety Monitoring and Reporting in non-therapeutic good trials. Participants do not give up any legal rights to compensation by participating in this study. If a participant suffers any injuries or complications as a result of the research project, they will be advised to contact the study team and will be assisted with arranging appropriate medical treatment.

Ethics and dissemination

The study will be performed according to the Declaration of Helsinki (2008) and the International Conference on Harmonisation - Good Clinical Practice (ICH-GCP) and has been reviewed and approved by the Human Research Ethics Committee (HREC) of the Sydney Local Health District (HREC Approval Number: X22-0042 & 2022/ETH0072, Protocol ID: BMC-YMH-003-2018, protocol version: V.3, 03/08/2022). The study has been registered in the Australian New Zealand Clinical Trial Registry (ACTRN12622000882729). Any amendments will be submitted to the HREC for review prior to implementation as per HREC guidelines.

The results of this study will be disseminated as widely as possible into the scientific and broader community, including publication in peer-reviewed journals, scholarly book chapters, presentation at conferences and publication in conference proceedings. This will include one paper investigating the primary outcome measure of this study (SOFAS scores), one paper determining the economic feasibility of the HP&MBC package, and a series of papers investigating secondary outcomes (e.g. depression, suicidality). For each paper, all authors will satisfy the Vancouver criteria for authorship.

Contributorship statement

IBH conceived the research idea and is the principal investigator. FI, AN, YJCS and NZ contributed to the study design and conception. CR, AN, YJCS and NZ wrote the study protocol with input from IBH, FI, WC, AJG, FML, JS, PM, CM, EK, MKC, SM, MA, CG, JC, DK, RB, BH, AL, MLH, DFH, MPC, HCC and ES. FI and CR wrote the manuscript with input from IBH, AN, NZ, YJCS, NZ, WC, AJG, FML, JS, PM, CM, EK, MKC, SM, MA, CG, JC, DK, RB, BH, AL, MLH, DFH and ES.

Acknowledgements

We are deeply indebted to all clinicians and health professionals at the participating centres who contribute to this clinical trial. Particularly to the Mind Plasticity Consortia which includes: Mark Yim, Bradley Whitwell and Alison Crowley; and the headspace Camperdown Consortia which includes Donna Fowler, Elana Volanakis, Vicki Papageorgopoulos, Matilda Russel and Elizabeth Phung.

Funding

For peer review only - http://bmjopen.bmj.com/site/about/guidelines.xhtml

This trial is an investigator-initiated trial funded by the NHMRC – 2020 Clinical Trials and Cohort Studies, Application ID: 2001568. Frank Iorfino is supported by the Bill and Patricia Ritchie Foundation Fellowship (ID: N/A). Jacob Crouse is supported by a NHMRC Emerging Leadership Fellowship (GNT2008197).

Data availability statement

Data generated from this study will not be published in an online repository.

Competing interests

A/Prof Elizabeth Scott is the Medical Director, Young Adult Mental Health Unit, St Vincent's Hospital Darlinghurst, Discipline Leader of Adult Mental Health, School of Medicine, University of Notre Dame, Research Affiliate, The University of Sydney and Consultant Psychiatrist. She has received honoraria for educational seminars related to the clinical management of depressive disorders supported by Servier and Eli-Lilly pharmaceuticals. She has participated in a national advisory board for the antidepressant compound Pristiq, manufactured by Pfizer. She was the National Coordinator of an antidepressant trial sponsored by Servier.

Professor Jan Scott is a visiting professor at Diderot University, the Norwegian University of Science and Technology, Swinburne University of Technology and The University of Sydney and a 'Science without Borders' fellow (Brazil). She has received grant funding from the UK Medical Research Council and from the UK Research for Patient Benefit programme; she declares no financial or other conflict of interests in relation to the topics addressed in this paper.

Professor Ian Hickie is the Co-Director, Health and Policy at the Brain and Mind Centre (BMC) University of Sydney, Australia. The BMC operates an early-intervention youth services at Camperdown under contract to headspace. Professor Hickie has previously led community-based and pharmaceutical industry-supported (Wyeth, Eli Lily, Servier, Pfizer, AstraZeneca) projects focused on the identification and better management of anxiety and depression. He is the Chief Scientific Advisor to, and a 3.2% equity shareholder in, Innowell Pty Ltd. Innowell was formed by the University of Sydney (45% equity) and PwC (Australia; 45% equity) to deliver the $30 M Australian Government-funded Project Synergy (2017-20) and to lead transformation of mental health services internationally through the use of innovative technologies.

All other authors declare no conflict of interest.

For peer review only - http://bmjopen.bmj.com/site/about/guidelines.xhtml

References

1. Bloom, D., et al., *The global economic burden of non-communicable diseases*. 2012, Program on the Global Demography of Aging.
2. Gustavsson, A., et al., *Cost of disorders of the brain in Europe 2010.* European Neuropsychopharmacology, 2011. **21**(10): p. 718-79.
3. Lopez, A.D., et al., *Global and regional burden of disease and risk factors, 2001: Systematic analysis of population health data.* The Lancet, 2006. **367**(9524): p. 1747-1757.
4. Merikangas, K.R., et al., *Lifetime prevalence of mental disorders in U.S. adolescents: results from the National Comorbidity Survey Replication--Adolescent Supplement (NCS-A).* J Am Acad Child Adolesc Psychiatry, 2010. **49**(10): p. 980-9.
5. Copeland, W.E., et al., *Adult Functional Outcomes of Common Childhood Psychiatric Problems: A Prospective, Longitudinal Study.* JAMA Psychiatry, 2015. **72**(9): p. 892-9.
6. Iorfino, F., et al., *Social and occupational outcomes for young people who attend early intervention mental health services: a longitudinal study.* Med J Aust, 2022. **216**(2): p. 87-93.
7. Iorfino, F., et al., *Delineating the trajectories of social and occupational functioning of young people attending early intervention mental health services in Australia: A longitudinal study.* BMJ Open, 2018. **8**(3): p. e020678.
8. Cross, S.P., D.F. Hermens, and I.B. Hickie, *Treatment patterns and short-term outcomes in an early intervention youth mental health service.* Early Intervention in Psychiatry, 2016. **10**(1): p. 88-97.
9. Hatfield, D., et al., *Do we know when our clients get worse? An investigation of therapists' ability to detect negative client change.* Clinical Psychology & Psychotherapy: An International Journal of Theory & Practice, 2010. **17**(1): p. 25-32.
10. Walfish, S., et al., *An investigation of self-assessment bias in mental health providers.* Psychological Reports, 2012. **110**(2): p. 639-644.
11. Scott, J., et al., *Pathways to depression by age 16 years: examining trajectories for self-reported psychological and somatic phenotypes across adolescence.* Journal of Affective Disorders, 2017.
12. Hansell, N.K., et al., *Genetic co-morbidity between neuroticism, anxiety/depression and somatic distress in a population sample of adolescent and young adult twins.* Psychological Medicine, 2012. **42**(6): p. 1249-1260.
13. Scott, J., et al., *Clinical staging in psychiatry: a cross-cutting model of diagnosis with heuristic and practical value.* The British Journal of Psychiatry, 2013. **202**(4): p. 243-245.
14. Cuthbert, B.N., *The RDoC framework: facilitating transition from ICD/DSM to dimensional approaches that integrate neuroscience and psychopathology.* World Psychiatry, 2014. **13**(1): p. 28-35.
15. Casey, B., et al., *DSM-5 and RDoC: progress in psychiatry research?* Nature Reviews Neuroscience, 2013. **14**(11): p. 810.
16. Ægisdóttir, S., et al., *The meta-analysis of clinical judgment project: Fifty-six years of accumulated research on clinical versus statistical prediction.* The Counseling Psychologist, 2006. **34**(3): p. 341-382.
17. Woodford, R., et al., *Accuracy of clinician predictions of future self-harm: a systematic review and meta-analysis of predictive studies.* Suicide and Life-Threatening Behavior, 2019. **49**(1): p. 23-40.

For peer review only - http://bmjopen.bmj.com/site/about/guidelines.xhtml

18. Hickie, I.B., et al., *Right care, first time: a highly personalised and measurement-based care model to manage youth mental health.* Medical Journal of Australia, 2019. **211**: p. S3-S46.
19. Iorfino, F., et al., *Multidimensional outcomes in youth mental health care: what matters and why?* MEDICAL JOURNAL OF AUSTRALIA, 2019. **211**: p. S4-S11.
20. Insel, T.R., *The arrival of preemptive psychiatry.* Early Intervention in Psychiatry, 2007. **1**(1): p. 5-6.
21. Insel, T.R., *Translating scientific opportunity into public health impact: A strategic plan for research on mental illness.* Archives of General Psychiatry, 2009. **66**(2): p. 128-133.
22. Scott, E.M., et al., *Targeted primary care-based mental health services for young Australians.* Medical Journal of Australia, 2012. **196**(2): p. 136-140.
23. Harding, K.J., et al., *Measurement-based care in psychiatric practice: a policy framework for implementation.* J Clin Psychiatry, 2011. **72**(8): p. 1136-43.
24. Goldberg, S.B., et al., *Measuring Psychiatric Symptoms Remotely: a Systematic Review of Remote Measurement-Based Care.* Curr Psychiatry Rep, 2018. **20**(10): p. 81.
25. Fortney, J.C., et al., *A Tipping Point for Measurement-Based Care.* Psychiatric Services, 2017. **68**(2): p. 179-188.
26. Waldrop, J. and T.M. McGuinness, *Measurement-Based Care in Psychiatry.* J Psychosoc Nurs Ment Health Serv, 2017. **55**(11): p. 30-35.
27. Hickie, I.B., *The role of new technologies in monitoring the evolution of psychopathology and providing measurement-based care in young people.* World Psychiatry, 2020. **19**(1): p. 38.
28. Lewis, C.C., et al., *Implementing measurement-based care (iMBC) for depression in community mental health: a dynamic cluster randomized trial study protocol.* Implementation Science, 2015. **10**(1): p. 127.
29. Scott, K. and C.C. Lewis, *Using measurement-based care to enhance any treatment.* Cognitive and behavioral practice, 2015. **22**(1): p. 49-59.
30. Bickman, L., et al., *Effects of routine feedback to clinicians on mental health outcomes of youths: Results of a randomized trial.* Psychiatric Services, 2011. **62**(12): p. 1423-1429.
31. Shimokawa, K., M.J. Lambert, and D.W. Smart, *Enhancing treatment outcome of patients at risk of treatment failure: meta-analytic and mega-analytic review of a psychotherapy quality assurance system.* Journal of consulting and clinical psychology, 2010. **78**(3): p. 298.
32. Tam, H. and K. Ronan, *The application of a feedback-informed approach in psychological service with youth: Systematic review and meta-analysis.* Clinical psychology review, 2017. **55**: p. 41-55.
33. Bauer, A.M., et al., *Comparing Approaches to Mobile Depression Assessment for Measurement-Based Care: Prospective Study.* Journal of Medical Internet Research, 2018. **20**(6): p. e10001.
34. Woltmann, E., et al., *Comparative effectiveness of collaborative chronic care models for mental health conditions across primary, specialty, and behavioral health care settings: systematic review and meta-analysis.* Am J Psychiatry, 2012. **169**(8): p. 790-804.
35. LaMonica, H.M., et al., *Informing the Future of Integrated Digital and Clinical Mental Health Care: Synthesis of the Outcomes From Project Synergy.* JMIR Mental Health, 2022. **9**(3): p. e33060.

For peer review only - http://bmjopen.bmj.com/site/about/guidelines.xhtml

36. Hickie, I.B., *Implementing 21st century "end-to-end" and technology-enhanced care for young people.* World Psychiatry, 2022. **21**(1): p. 79.
37. Goldman, H.H., A.E. Skodol, and T.R. Lave, *Revising axis V for DSM-IV: a review of measures of social functioning.* Am J Psychiatry, 1992. **149**(9): p. 1148-1156.
38. Cross, S.P., et al., *Variability in Clinical Outcomes for Youths Treated for Subthreshold Severe Mental Disorders at an Early Intervention Service.* Psychiatr Serv, 2018. **69**(5): p. 555-561.
39. Iorfino, F., et al., *A Digital Platform Designed for Youth Mental Health Services to Deliver Personalized and Measurement-Based Care.* Frontiers in Psychiatry, 2019. **10**.
40. Fowler, D., et al., *Social recovery therapy in combination with early intervention services for enhancement of social recovery in patients with first-episode psychosis (SUPEREDEN3): a single-blind, randomised controlled trial.* Lancet Psychiatry, 2018. **5**(1): p. 41-50.
41. Scaini, S., et al., *A comprehensive meta-analysis of cognitive-behavioral interventions for social anxiety disorder in children and adolescents.* Journal of Anxiety Disorders, 2016. **42**: p. 105-112.
42. Carpenter, J.K., et al., *Cognitive behavioral therapy for anxiety and related disorders: A meta-analysis of randomized placebo-controlled trials.* Depress Anxiety, 2018. **35**(6): p. 502-514.
43. Robillard, R., et al., *Parallel Changes in Mood and Melatonin Rhythm Following an Adjunctive Multimodal Chronobiological Intervention With Agomelatine in People With Depression: A Proof of Concept Open Label Study.* Front Psychiatry, 2018. **9**: p. 624.
44. Wu, J.Q., et al., *Cognitive Behavioral Therapy for Insomnia Comorbid With Psychiatric and Medical Conditions: A Meta-analysis.* JAMA Intern Med, 2015. **175**(9): p. 1461-72.
45. Cunningham, J.E.A., J.A. Stamp, and C.M. Shapiro, *Sleep and major depressive disorder: a review of non-pharmacological chronotherapeutic treatments for unipolar depression.* Sleep Med, 2019.
46. Kopelowicz, A., R.P. Liberman, and R. Zarate, *Recent advances in social skills training for schizophrenia.* Schizophr Bull, 2006. **32 Suppl 1**(Suppl 1): p. S12-23.
47. Eisner, L., et al., *Dialectical Behavior Therapy Group Skills Training for Bipolar Disorder.* Behav Ther, 2017. **48**(4): p. 557-566.
48. Ritschel, L.A., N.E. Lim, and L.M. Stewart, *Transdiagnostic Applications of DBT for Adolescents and Adults.* Am J Psychother, 2015. **69**(2): p. 111-28.
49. McGorry, P. and I.B. Hickie, *Clinical Staging in Psychiatry: Making Diagnosis Work for Research and Treatment*. 2019, Cambridge: Cambridge University Press.
50. Lee, Y.Y., et al., *Economic evaluation of the Target-D platform to match depression management to severity prognosis in primary care: A within-trial cost-utility analysis.* PloS one, 2022. **17**(5): p. e0268948.
51. Chatterton, M.L., et al., *Economic evaluation of stepped care for the management of childhood anxiety disorders: Results from a randomised trial.* Australian & New Zealand Journal of Psychiatry, 2019. **53**(7): p. 673-682.
52. Keetharuth, A.D., et al., *Recovering Quality of Life (ReQoL): a new generic self-reported outcome measure for use with people experiencing mental health difficulties.* The British Journal of Psychiatry, 2018. **212**(1): p. 42-49.
53. Keetharuth, A.D., et al., *Estimating a preference-based index for mental health from the recovering quality of life measure: valuation of recovering quality of life utility index.* Value in Health, 2021. **24**(2): p. 281-290.

For peer review only - http://bmjopen.bmj.com/site/about/guidelines.xhtml

54. Reeve, R., *Confidence interval of difference of proportions in logistic regression in presence of covariates.* Statistical methods in medical research, 2018. **27**(2): p. 451-465.

For peer review only

For peer review only - http://bmjopen.bmj.com/site/about/guidelines.xhtml

Figure Legends

Figure 1: Study design and service subgroups. An overview of how the trial design gives rise to distinct groups within a single participating service. There are two implementation phases and three research arms associated with this trial which result in four distinct groups for each service based on a young person's exposure and trial participation status. Group 1 is used to establish baseline outcome statistics for the service prior to the trial commencing. Groups 2, 3 and 4 differ based on the trial status which will determine what treatments they receive. The primary outcome analysis for the RCT will be between groups 3 and 4. Routine outcome evaluation data collection is ongoing from the first phase of the trial whereby all groups will be followed up using the same processes and practices. BAU= Business as usual.

Figure 2: Study flow diagram (CONSORT style)

Figure 3. An example dashboard of results from the Innowell Platform

For peer review only

For peer review only - http://bmjopen.bmj.com/site/about/guidelines.xhtml

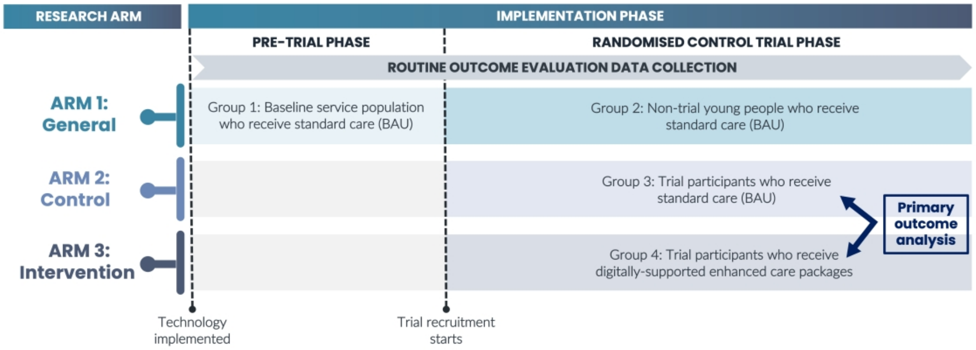

Figure 1: An overview of how the trial design gives rise to distinct groups within a single participating service. There are two implementation phases and three research arms associated with this trial which result in four distinct groups for each service based on a young person's exposure and trial participation status. Group 1 is used to establish baseline outcome statistics for the service prior to the trial commencing. Groups 2, 3 and 4 differ based on the trial status which will determine what treatments they receive. The primary outcome analysis for the RCT will be between groups 3 and 4. Routine outcome evaluation data collection is ongoing from the first phase of the trial whereby all groups will be followed up using the same processes and practices. BAU= Business as usual.

296x108mm (400 x 400 DPI)

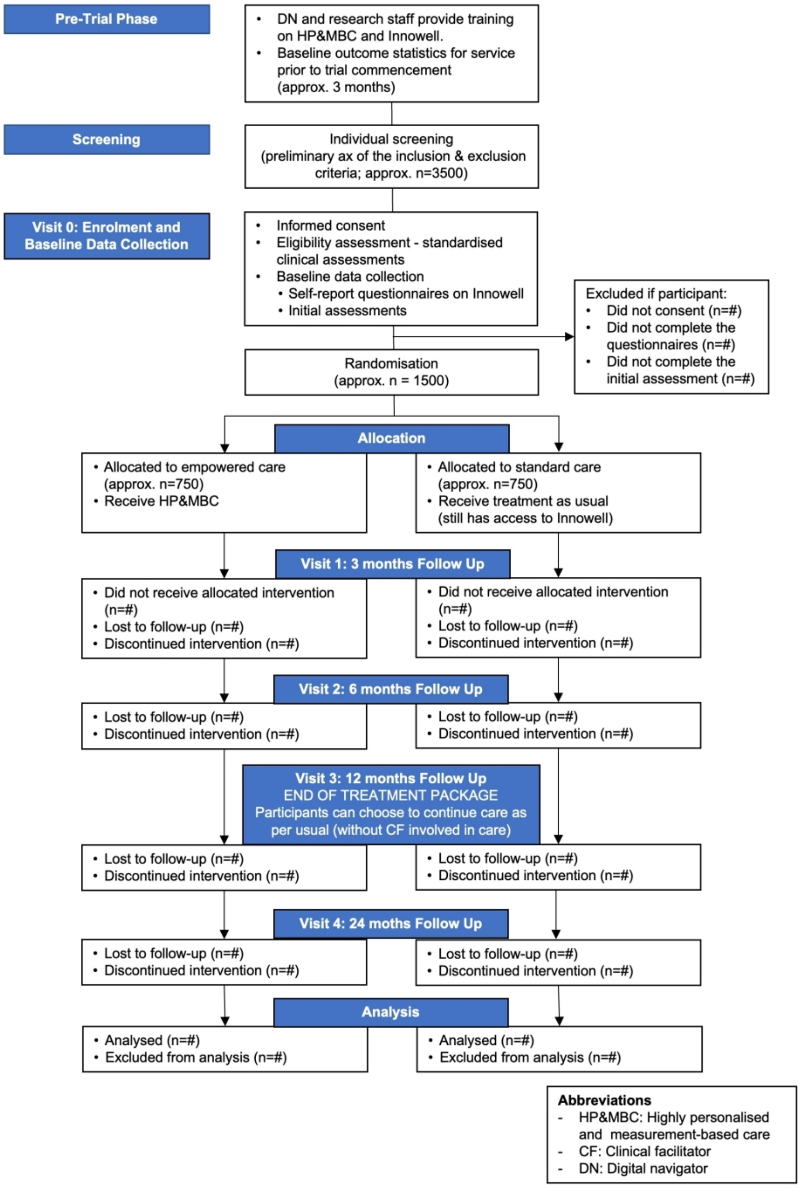

Figure 2: Study flow diagram (CONSORT style)

87x129mm (500 x 500 DPI)

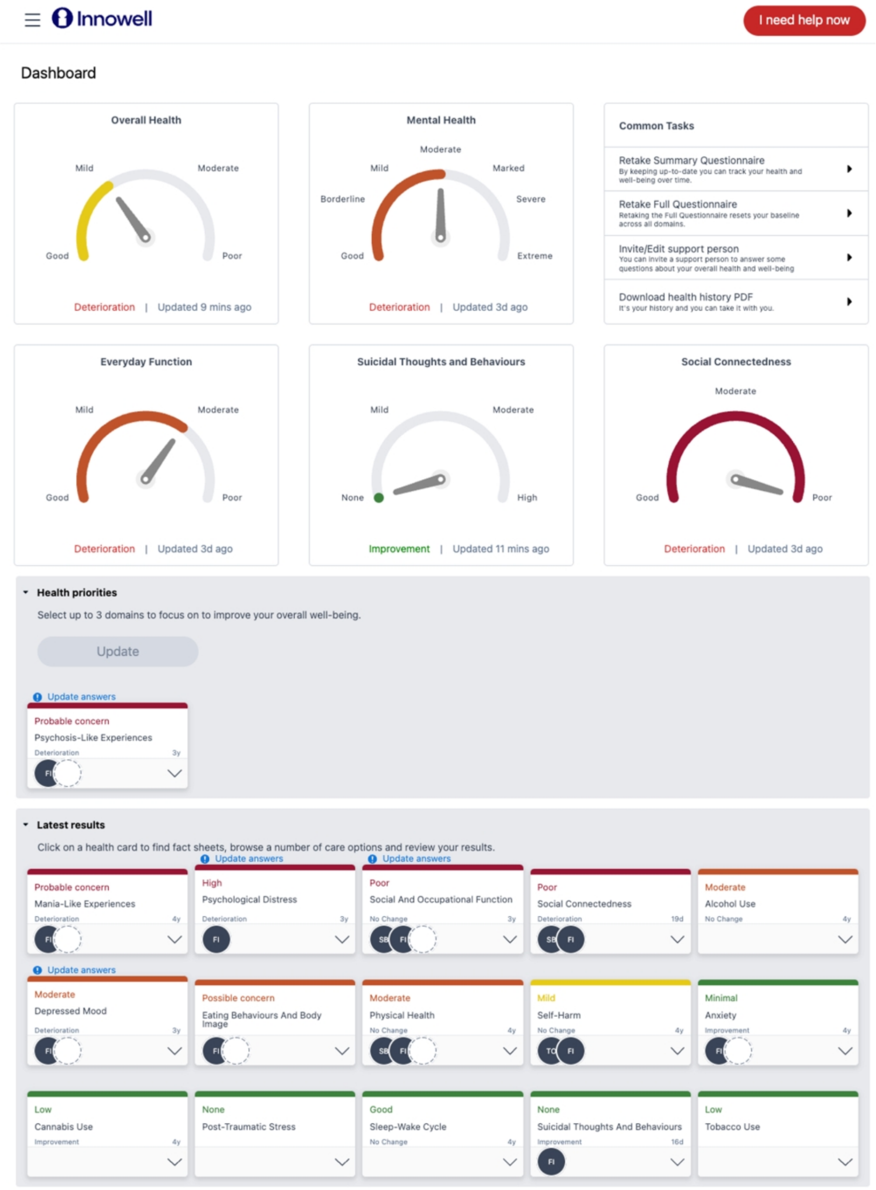

Figure 3. An example dashboard of results from the Innowell Platform

108x147mm (500 x 500 DPI)

THE UNIVERSITY OF SYDNEY

Brain and Mind Centre

Participant Information Sheet/Consent Form

Interventional Study - *Adult providing own consent*

Mind Plasticity – Brain and Mind Centre

**Title**	A large-scale clinical effectiveness (health services) trial to determine whether personalised health care packages, combined with digitally-supported measurement-based care, improve functional outcomes in young people with mood disorders.
**Short Title**	EMPOWERED Trial.
**Protocol Number**	BMC-YMH-003-2018
**Project Sponsor**	The University of Sydney
**Coordinating Principal Investigator/ Principal Investigator**	Professor Ian B. Hickie
**Location**	Brain and Mind Centre, University of Sydney<br>Mind Plasticity, Surry Hills

Part 1 What does my participation involve?

1 Introduction

You are invited to take part in this research project which is investigating whether more personalised health care packages, linked with continuous digital feedback, deliver better functional improvements at 12 months (and follow-up for a further 12 months after cessation of active care) than digitally-supported assessment linked to standard care packages.

This Participant Information Sheet (PIS) tells you about the research project. It explains the tests and treatments involved. Knowing what is involved will help you decide if you want to take part in the research.

Please read this information carefully. Ask questions about anything that you don't understand or want to know more about. Before deciding whether or not to take part, you might want to talk about it with a relative, friend, or your local doctor.

Participation in this research is voluntary. If you don't wish to take part, you don't have to. You will receive the best possible care whether or not you take part.

For peer review only - http://bmjopen.bmj.com/site/about/guidelines.xhtml

If you decide you want to take part in the research project, you will be asked to sign the consent section. By signing it you are telling us that you:

- Understand what you have read
- Consent to take part in the research project
- Consent to have the tests and treatments that are described
- Consent to the use of your personal and health information as described.

You will be given a copy of this Participant Information and Consent Form to keep.

2 What is the purpose of this research?

This study aims to assess in 1500 young people with mood syndromes, whether more personalised health care packages, linked with continuous digital feedback (i.e. highly personalised and measurement based care (HP&MBC), deliver better functional improvements at 12 months (and follow-up for a further 12 months after cessation of active care), than digitally-supported assessment linked to standard care packages.

Although the standard care packages are an improved offering (through greater standardisation of assessment and feedback of those assessments), we hypothesise that the HP&MBC treatment packages are superior, by implementing continuous monitoring and care coordination through the use of digital technologies, and providing extensive feedback to the clinical service, the treating clinician, and the young person and their family or carer. The continuous feedback will detect unmet care, increase the likelihood of identifying young persons that do not respond to treatment, and facilitate the process to optimise care and increase the engagement of young people in their own care.

This research has been initiated by the investigator, Professor Ian B. Hickie, Co-Director and Consultant Psychiatrist, Brain and Mind Centre, The University of Sydney. This research is being conducted by the Brain and Mind Centre and Mind Plasticity. The study has been funded by NHMRC 2020 Clinical Trials and Cohort Studies (Application ID: 2001568).

3 What does participation in this research involve?

If you consent to participate, you will be taking part in a randomised controlled research project. Sometimes we do not know which treatment is best for treating a condition. To find out we need to compare different treatments. We put people into groups and give each group a different treatment. The results are compared to see if one is better. To try to make sure the groups are the same, each participant is put into a group by chance (at random).

You have been invited to take part in this trial as your clinician indicated that you may be eligible and interested in taking part. This clinical trial comprises 12-months of an active treatment care package and a 12-month follow-up phase, meaning the duration of the trial is expected to be 24 months from your baseline visit. If you decide to take part, an appointment will be scheduled for an enrolment visit at the Brain and Mind Centre (BMC).

Enrolment Visit *(time commitment: 60 minutes)*

During your enrolment visit, a staff member will confirm that you have read and understood this PIS, the study will also be verbally explained to you, and you will be given the opportunity to ask any questions you may have. If you consent to participating you will be asked to sign a written consent form.

For peer review only - http://bmjopen.bmj.com/site/about/guidelines.xhtml

Once your consent is provided, you will be asked some relevant questions to confirm that you meet the other inclusion and exclusion criteria for the study. If you are eligible to take part you will be invited to attend a baseline visit.

Once enrolled in the study, you will be randomly assigned to receive one of two treatment packages for a 12 month duration.

You will have approximately equal chance of being assigned to one of the following two care packages:

1. The *Highly Personalised and Measurement Based Care (HP&MBC)* Package: This includes:
   - Initial e-health assessment with feedback provided to yourself, your treating clinician, and the clinical service.
   - Continuous monitoring via e-health assessment and monthly feedback over the 12-month treatment duration to yourself, your treating clinician, and the clinical service.
   - Personalised referrals over the 12-month treatment duration, to specific treatment programs that may be beneficial to you,based on the outcome of continuous assessment data.

2. *Standardised care package:* This includes:
   - Initial e-health assessment with feedback provided to yourself, your treating clinician, and the clinical service.
   - Provision of standard multidisciplinary care options and ongoing access to other relevant psychological and pharmacological options.
   - Additional e-health assessments at 3, 6 and 12-months.

Baseline Visit *(time commitment: 60-90 minutes)*

During your baseline visit, you will be asked questions about your day-to-day activities, mood, and behaviour.

You will also be asked to complete a series of online self-report assessments that will include further questions about your mental health symptoms, physical activity & physical health, sleep, and the quality of your relationships and social supports. These self-report questions will be accessed online and completed via iPad.

After the baseline assessment, you will be required to visit your local pathology centre for a blood test for the assessment of metabolic, inflammatory, and standard blood markers. The results will be sent to the study doctor.

Follow-up assessments (3, 6, 12, 24 months) (*time commitment: 60-90 minutes)*

You will be invited back for follow-up research assessments 3, 6, 12 and 24 months after the commencement of your treatment. Interview and online questionnaire assessments will again be conducted, including questions about your day-to-day activities, mood, and behaviour.

At the **6 & 12-month** time points you will also be required to visit your local pathology centre for a blood test which will again be sent to the study doctor.

For peer review only - http://bmjopen.bmj.com/site/about/guidelines.xhtml

Additional Costs

Throughout the research project specific treatment programs may be recommended to you based on the outcome of continuous assessment data. There may be additional costs associated with these treatment options. Any additional costs will be discussed with you in advance, and you will have the option to decline.

If you have a local doctor, we recommend that you inform them of your participation in this research project. In addition, the researchers would like to have access to your medical record to obtain information relevant to this study.

You will be reimbursed for your time in this study. Reimbursement will be $50 and this will be provided to you in the form of a Coles/Myer voucher.

4 What do I have to do?

To participate in this study you must meet some criteria, including:

- Aged 15-25 years old, seeking help for psychological distress
- Classified as suitable for the intervention based on the enrolment assessment
- Written informed consent

You will *not* be able to participate in the study if you meet certain criteria, including:

- Acute suicidal or aggressive behaviour requiring alternative care
- Depressive syndrome secondary to a primary medical condition
- Intellectual disability

5 Other relevant information about the research project

This study will involve 1500 participants, and will primarily be conducted at The Brain and Mind Centre, University of Sydney, 94-100 Mallet Street, Camperdown and Mind Plasticity, Suite 517, Level 5, 50 Holt St, Surry Hills NSW 2010.

6 Do I have to take part in this research project?

Participation in any research project is voluntary. If you do not wish to take part, you do not have to. If you decide to take part and later change your mind, you are free to withdraw from the project at any stage; you don't have to give a reason. If you do want to take part now, but change our mind later, you can pull out of the study at any time.

If you do decide to take part, you will be given this Participant Information and Consent Form to sign and you will be given a copy to keep.

For peer review only - http://bmjopen.bmj.com/site/about/guidelines.xhtml

Your decision whether to take part or not to take part, or to take part and then withdraw, will not affect your relationship with those treating you or your relationship with the Mind Plasticity and/or Brain and Mind Centre, The University of Sydney.

7 What are the alternatives to participation?

You do not have to take part in this research project to receive treatment. Participation in this research is not your only option. Other options are available; these include engaging in / continuing with the standard treatment options offered. The study doctor will discuss these options with you before you decide whether or not to take part in this research project. You can also discuss the options with your local doctor and/or treating clinician.

8 What are the possible benefits of taking part?

We cannot guarantee or promise that you will receive any direct benefits from being in this study. However, the aim of this study is to further the knowledge and treatment of mood disorders in young people.

9 What are the possible risks and disadvantages of taking part?

We anticipate that the possible risks associated with this study are no more than low risk. You may feel anxious during some of the assessment procedures. If you become upset or distressed as a result of your participation in the research, the study doctor will be able to arrange for counselling or other appropriate support. Any counselling or support will be provided by a qualified staff who are not members of the research project team.

Having a blood sample taken may cause some discomfort, bruising, minor infection or bleeding. If this happens, it can be easily treated by your local doctor.

The study doctor will review the results of your blood test. In the event that the results of your blood test require further investigation, you will be referred to your local doctor.

10 What will happen to my test samples?

You will be asked to provide blood samples at baseline, 6 and 12 months post baseline. Blood samples will be collected in a fasting state by a trained phlebotomist at your local pathology centre. Standard infection control procedures will be followed to avoid harm to participants. Blood samples will be used to identify the following:

- Metabolic blood measures
- Inflammatory markers
- Standard clinical blood measures

All blood test results will be labelled with your study ID code only, and no identifying information.

Your data can only be obtained and used by researchers who have their study approved by a Human Research Ethics Committee. Any scientists who wish to use your data must also agree to protect your privacy and store data securely.

All blood test results will be reviewed by the study doctor. If there are any abnormalities identified in the blood results, you will be notified and asked to see your local treating GP for

For peer review only - http://bmjopen.bmj.com/site/about/guidelines.xhtml

review. Blood samples will not be stored as part of this study and therefore samples will not be retained for future use.

11 What if new information arises during this research project?

Sometimes during the course of a research project, new information becomes available about the treatment that is being studied. If this happens, your study doctor will tell you about it and discuss with you whether you want to continue in the research project. If you decide to withdraw, your study doctor will make arrangements for your regular health care to continue. If you decide to continue in the research project you will be asked to sign an updated consent form.

Also, on receiving new information, your study doctor might consider it to be in your best interests to withdraw you from the research project. If this happens, he/ she will explain the reasons and arrange for your regular health care to continue.

12 What if I withdraw from this research project?

You can withdraw from the study at any time by contacting research staff. Your decision whether to participate will not affect your current or future relationship with the researchers or anyone else at The University of Sydney nor your current or future involvement with the mental health service.

If you decide to withdraw from the project, please notify a member of the research team before you withdraw. This notice will allow that person or the research supervisor to discuss any health risks or special requirements linked to withdrawing.

You will be asked about the reason(s) for your discontinuation and about the presence of any adverse events. You will be invited to attend the Brain and Mind Centre for a final close-out visit to complete all assessments normally completed at the final study visit (i.e., 12-months post baseline).

Further, relevant information about your health status as judged by the investigator, which comes up after the screening visit may justify a subsequent exclusion from the study. Relevant information could be information concerning inclusion or exclusion criteria or information that implies that the treatment schedule is not suitable.

If you do withdraw your consent during the research project, relevant study staff will not collect additional personal information from you, although personal information already collected will be retained to ensure that the results of the research project can be measured properly and to comply with law. You should be aware that data collected up to the time you withdraw will form part of the research project results. If you do not want them to do this, you must tell them before you join the research project.

13 Could this research project be stopped unexpectedly?

In the unlikely event that the local regulatory/health authorities suspend the trial, you would still be able to access your treatment as usual. It won't impact your relationship with the University of Sydney or the service from which you are accessing care.

For peer review only - http://bmjopen.bmj.com/site/about/guidelines.xhtml

Part 2 How is the research project being conducted?

14 What will happen to information about me?

By signing the consent form you consent to the study doctor and relevant research staff collecting and using personal information about you for the research project. Any information obtained in connection with this research project that can identify you will remain confidential. Your information will only be used for the purpose of this research project and it will only be disclosed with your permission, except as required by law.

Information about you may be obtained from your health records held at this and other health services for the purpose of this research. By signing the consent form you agree to the study team accessing health records if they are relevant to your participation in this research project.

Your health records and any information obtained during the research project are subject to inspection (for the purpose of verifying the procedures and the data) by the relevant authorities and authorised representatives of the Sponsor, The University of Sydney, or as required by law. By signing the Consent Form, you authorise release of, or access to, this confidential information to the relevant study personnel and regulatory authorities as noted above.

It is anticipated that the results of this research project will be published and/or presented in a variety of forums. In any publication and/or presentation, information will be provided in such a way that you cannot be identified, except with your permission.

Information about your participation in this research project may be recorded in your health records.

In accordance with relevant Australian and NSW privacy and other relevant laws, you have the right to request access to your information collected and stored by the research team. You also have the right to request that any information with which you disagree be corrected. Please contact the study team member named at the end of this document if you would like to access your information.

Any information obtained for the purpose of this research project and for the future research described in Section 14 that can identify you will be treated as confidential and securely stored. It will be disclosed only with your permission, or as required by law.

All study records will be stored for a minimum of 20 years post study completion and then securely destroyed.

All data collected for the purposes of the study will be linked to unique study ID codes and will not contain identifying information. Data will be stored separately from any identifying information (e.g., signed consent forms). One senior research staff member at each site will have an electronic password protected file linking participant names and identification codes (i.e., data will be re-identifiable). Individuals will not be named in any reports or publications resulting from the study, and no document containing identifying information will leave the study site. Any publications based on the study will include only pooled results from participants.

Data collection will be conducted only by authorised members of study staff, to whom this duty has been allocated and who are named on the Human Ethics application and Governance approvals for the trial. Only sufficiently trained and supervised research staff will be delegated to enter and analyse data. We will be using a RedCap database to enter the research data. Data for which hardcopies are generated will be stored in both original hard copy and electronic form. This data will be kept under 1) lock and key at trial site or 2) electronic file that is password

For peer review only - http://bmjopen.bmj.com/site/about/guidelines.xhtml

protected and accessible only by research staff responsible for data entry or monitoring. Electronic data generated by participant outcomes will be electronically stored via the *Innowell Online Platform*. The information stored via this online platform will be de-identified and subject to privacy policies and the Research Code of Conduct. All data will be analysed by study staff at the Brain and Mind Centre, The University of Sydney. There will be no sharing or pooling of data with other collaborators.

With consent, we may use data collected in this study for future research purposes.

15 Complaints and compensation

If you suffer any injuries or complications as a result of this research project, you should contact the study team as soon as possible and you will be assisted with arranging appropriate medical treatment. If you are eligible for Medicare, you can receive any medical treatment required to treat the injury or complication, free of charge, as a public patient in any Australian public hospital.

In addition, you may have a right to take legal action to obtain compensation for any injuries or complications resulting from the study. Compensation may be available if your injury or complication is sufficiently serious and is caused by unsafe equipment, or by the negligence of one of the parties involved in the study (for example, the researcher, the clinic, or the treating clinician). You do not give up any legal rights to compensation by participating in this study.

16 Who is organising and funding the research?

This research project is being conducted by Professor Ian Hickie at The Brain and Mind Centre, Sydney University.

This trial is an investigator-initiated trial funded by the NHMRC – 2020 Clinical Trials and Cohort Studies, Application ID: 2001568.

You will not benefit financially from your involvement in this research project.

No member of the research team will receive a personal financial benefit from your involvement in this research project (other than their ordinary wages).

17 Who has reviewed the research project?

All research in Australia involving humans is reviewed by an independent group of people called a Human Research Ethics Committee (HREC). The ethical aspects of this research project have been approved by the HREC of the Sydney Local Health District (RPAH Zone). This research project has been designed to make sure the researchers interpret the results in a fair and appropriate way and avoid study researchers or participants jumping to conclusions.

This project will be carried out according to the *National Statement on Ethical Conduct in Human Research (2007)*. This statement has been developed to protect the interests of people who agree to participate in human research studies.

18 Further information and who to contact

The person you may need to contact will depend on the nature of your query.

For peer review only - http://bmjopen.bmj.com/site/about/guidelines.xhtml

Clinical contact person

Name	Professor Ian Hickie
Position	Principal Investigator
Telephone	(02) 93510810
Email	Ian.hickie@sydney.edu.au

For matters relating to research at the site at which you are participating, the details of the local site complaints person are:

Complaints contact person/s

Name	Ms Alissa Nichles, Ms Natalia Zmicerevska
Position	Senior Clinical research Officers
Telephone	(02) 9114 4100
Email	Alissa.nichles@sydney.edu.au, Natalia.zmicerevska@sydney.edu.au

If you have any complaints about any aspect of the project, the way it is being conducted or any questions about being a research participant in general, then you may contact:

This study has been approved by the Ethics Review Committee (RPAH Zone) of the Sydney Local Health District. Any person with concerns or complaints about the conduct of this study should contact the Executive Officer on 02 9515 6766 and quote Protocol number X22-0042.

For peer review only - http://bmjopen.bmj.com/site/about/guidelines.xhtml

Consent Form - *Adult providing own consent*

**Title**	A large-scale clinical effectiveness (health services) trial to determine whether personal-ised health care packages, combined with digitally-supported measurement-based care, improve functional outcomes in young people with mood disorders.
**Short Title**	EMPOWERED Trial.
**Protocol Number**	BMC-YMH-003-2018
**Project Sponsor**	The University of Sydney
**Coordinating Principal Investigator/ Principal Investigator**	Professor Ian B. Hickie
**Location**	Brain and Mind Centre, University of Sydney<br>Mind Plasticity, Surry Hills

Declaration by Participant

I have read the Participant Information Sheet or someone has read it to me in a language that I understand.

I understand the purposes, procedures and risks of the research described in the project.

I give permission for my doctors, other health professionals, hospitals or laboratories outside this hospital to release information to Brain and Mind Centre, University of Sydney concerning my disease and treatment for the purposes of this project. I understand that such information will remain confidential.

I understand that my participation in this study will allow the researchers and others, as described in the Information for Participants, to have access to my medical record, and I agree to this.

I have had an opportunity to ask questions and I am satisfied with the answers I have received.

I freely agree to participate in this research project as described and understand that I am free to withdraw at any time during the study without affecting my future health care.

I understand that I will be given a signed copy of this document to keep.

I understand that, if I decide to discontinue the study treatment, I may be asked to attend follow-up visits to allow collection of information regarding my health status. Alternatively, a member of the research team may request my permission to obtain access to my medical records for collection of follow-up information for the purposes of research and analysis.

	Consent of participant
In addition, I also give consent for my health information to be used for future research purposes:	☐ Yes ☐ No
I give permission for the research data collected about me in this study, to be linked with data from any other research study I participate in that is run as part of the Youth Mental Health Research Program led by Professor Ian Hickie.	☐ Yes ☐ No

For peer review only - http://bmjopen.bmj.com/site/about/guidelines.xhtml

I would like to receive an overall summary of the results of this current study (via newsletter) once they are made available:	If yes, please provide email address: ______________@____________________

Name of Participant (please print) ______________________________________

Signature ______________________________ Date ______________________________

Name of Witness* to Participant's Signature (please print) ______________________________________

Signature ______________________________ Date ______________________________

* Witness is not to be the investigator, a member of the study team or their delegate. In the event that an interpreter is used, the interpreter may not act as a witness to the consent process. Witness must be 18 years or older.

Declaration by Study Doctor/Senior Researcher†

I have given a verbal explanation of the research project, its procedures and risks and I believe that the participant has understood that explanation.

Name of Study Doctor/ Senior Researcher† (please print) ______________________________________

Signature ______________________________ Date ______________________________

† A senior member of the research team must provide the explanation of, and information concerning, the research project.

Note: All parties signing the consent section must date their own signature.

For peer review only - http://bmjopen.bmj.com/site/about/guidelines.xhtml

Form for Withdrawal of Participation

**Title**	A large-scale clinical effectiveness (health services) trial to determine whether personalised health care packages, combined with digitally-supported measurement-based care, improve functional outcomes in young people with mood disorders.
**Short Title**	EMPOWERED Trial.
**Protocol Number**	BMC-YMH-003-2018
**Project Sponsor**	The University of Sydney
**Principal Investigator**	Professor Ian B. Hickie
**Location**	Brain and Mind Centre, University of Sydney<br>Mind Plasticity, Surry Hills

For peer review only

Declaration by Participant

I wish to withdraw from participation in the above research project and understand that such withdrawal will not affect my routine treatment, my relationship with those treating me or my relationship with Brain and Mind Centre/Mind Plasticity.

Name of Participant (please print) ______________________________

Signature ______________________ Date ______________________

IF NECESSARY: Description of circumstances of withdrawal below (to be written by Study Doctor/Senior Researcher)

Declaration by Study Doctor/Senior Researcher†

I have given a verbal explanation of the implications of withdrawal from the research project and I believe that the participant has understood that explanation.

Name of Study Doctor/
Senior Researcher† (please print) ______________________________

For peer review only - http://bmjopen.bmj.com/site/about/guidelines.xhtml

Signature ________________________________ Date________________________________

[†] A senior member of the research team must provide the explanation of and information concerning withdrawal from the research project.

Note: All parties signing the consent section must date their own signature.

For peer review only

For peer review only - http://bmjopen.bmj.com/site/about/guidelines.xhtml

SPIRIT
STANDARD PROTOCOL ITEMS: RECOMMENDATIONS FOR INTERVENTIONAL TRIALS

SPIRIT 2013 Checklist: Recommended items to address in a clinical trial protocol and related documents*

The EMPOWERED trial: Protocol for a randomised control trial of digitally supported, highly personalised and measurement-based care to improve functional outcomes in young people with mood disorders

Section/item	Item No	Description	Page
**Administrative information**			
Title	1	Descriptive title identifying the study design, population, interventions, and, if applicable, trial acronym	1
Trial registration	2a	Trial identifier and registry name. If not yet registered, name of intended registry	2
	2b	All items from the World Health Organization Trial Registration Data Set	1-17
Protocol version	3	Date and version identifier	2
Funding	4	Sources and types of financial, material, and other support	17-18
Roles and responsibilities	5a	Names, affiliations, and roles of protocol contributors	1, 17-18
	5b	Name and contact information for the trial sponsor	1
	5c	Role of study sponsor and funders, if any, in study design; collection, management, analysis, and interpretation of data; writing of the report; and the decision to submit the report for publication, including whether they will have ultimate authority over any of these activities	17
	5d	Composition, roles, and responsibilities of the coordinating centre, steering committee, endpoint adjudication committee, data management team, and other individuals or groups overseeing the trial, if applicable (see Item 21a for data monitoring committee)	NA
**Introduction**			
Background and rationale	6a	Description of research question and justification for undertaking the trial, including summary of relevant studies (published and unpublished) examining benefits and harms for each intervention	4-5
	6b	Explanation for choice of comparators	4-5
Objectives	7	Specific objectives or hypotheses	4-5

For peer review only

Trial design	8	Description of trial design including type of trial (eg, parallel group, crossover, factorial, single group), allocation ratio, and framework (eg, superiority, equivalence, noninferiority, exploratory)	4-5

Methods: Participants, interventions, and outcomes

Study setting	9	Description of study settings (eg, community clinic, academic hospital) and list of countries where data will be collected. Reference to where list of study sites can be obtained	5
Eligibility criteria	10	Inclusion and exclusion criteria for participants. If applicable, eligibility criteria for study centres and individuals who will perform the interventions (eg, surgeons, psychotherapists)	6-7
Interventions	11a	Interventions for each group with sufficient detail to allow replication, including how and when they will be administered	6-12
	11b	Criteria for discontinuing or modifying allocated interventions for a given trial participant (eg, drug dose change in response to harms, participant request, or improving/worsening disease)	NA (Not relevant)
	11c	Strategies to improve adherence to intervention protocols, and any procedures for monitoring adherence (eg, drug tablet return, laboratory tests)	10-12
	11d	Relevant concomitant care and interventions that are permitted or prohibited during the trial	9-10
Outcomes	12	Primary, secondary, and other outcomes, including the specific measurement variable (eg, systolic blood pressure), analysis metric (eg, change from baseline, final value, time to event), method of aggregation (eg, median, proportion), and time point for each outcome. Explanation of the clinical relevance of chosen efficacy and harm outcomes is strongly recommended	12-14
Participant timeline	13	Time schedule of enrolment, interventions (including any run-ins and washouts), assessments, and visits for participants. A schematic diagram is highly recommended (see Figure)	5
Sample size	14	Estimated number of participants needed to achieve study objectives and how it was determined, including clinical and statistical assumptions supporting any sample size calculations	6
Recruitment	15	Strategies for achieving adequate participant enrolment to reach target sample size	10-12

Methods: Assignment of interventions (for controlled trials)

Allocation:

Sequence generation	16a	Method of generating the allocation sequence (eg, computer-generated random numbers), and list of any factors for stratification. To reduce predictability of a random sequence, details of any planned restriction (eg, blocking) should be provided in a separate document that is unavailable to those who enrol participants or assign interventions	7
Allocation concealment mechanism	16b	Mechanism of implementing the allocation sequence (eg, central telephone; sequentially numbered, opaque, sealed envelopes), describing any steps to conceal the sequence until interventions are assigned	7
Implementation	16c	Who will generate the allocation sequence, who will enrol participants, and who will assign participants to interventions	7
Blinding (masking)	17a	Who will be blinded after assignment to interventions (eg, trial participants, care providers, outcome assessors, data analysts), and how	5, 7
	17b	If blinded, circumstances under which unblinding is permissible, and procedure for revealing a participant's allocated intervention during the trial	Na
**Methods: Data collection, management, and analysis**			
Data collection methods	18a	Plans for assessment and collection of outcome, baseline, and other trial data, including any related processes to promote data quality (eg, duplicate measurements, training of assessors) and a description of study instruments (eg, questionnaires, laboratory tests) along with their reliability and validity, if known. Reference to where data collection forms can be found, if not in the protocol	12-14
	18b	Plans to promote participant retention and complete follow-up, including list of any outcome data to be collected for participants who discontinue or deviate from intervention protocols	10-12
Data management	19	Plans for data entry, coding, security, and storage, including any related processes to promote data quality (eg, double data entry; range checks for data values). Reference to where details of data management procedures can be found, if not in the protocol	16
Statistical methods	20a	Statistical methods for analysing primary and secondary outcomes. Reference to where other details of the statistical analysis plan can be found, if not in the protocol	15-16
	20b	Methods for any additional analyses (eg, subgroup and adjusted analyses)	15-16
	20c	Definition of analysis population relating to protocol non-adherence (eg, as randomised analysis), and any statistical methods to handle missing data (eg, multiple imputation)	15-16

Methods: Monitoring

Data monitoring	21a	Composition of data monitoring committee (DMC); summary of its role and reporting structure; statement of whether it is independent from the sponsor and competing interests; and reference to where further details about its charter can be found, if not in the protocol. Alternatively, an explanation of why a DMC is not needed	NA (not a TGA reportable drug trial)
	21b	Description of any interim analyses and stopping guidelines, including who will have access to these interim results and make the final decision to terminate the trial	NA (not a TGA reportable drug trial)
Harms	22	Plans for collecting, assessing, reporting, and managing solicited and spontaneously reported adverse events and other unintended effects of trial interventions or trial conduct	16-17
Auditing	23	Frequency and procedures for auditing trial conduct, if any, and whether the process will be independent from investigators and the sponsor	16

Ethics and dissemination

Research ethics approval	24	Plans for seeking research ethics committee/institutional review board (REC/IRB) approval	17
Protocol amendments	25	Plans for communicating important protocol modifications (eg, changes to eligibility criteria, outcomes, analyses) to relevant parties (eg, investigators, REC/IRBs, trial participants, trial registries, journals, regulators)	17
Consent or assent	26a	Who will obtain informed consent or assent from potential trial participants or authorised surrogates, and how (see Item 32)	7
	26b	Additional consent provisions for collection and use of participant data and biological specimens in ancillary studies, if applicable	na
Confidentiality	27	How personal information about potential and enrolled participants will be collected, shared, and maintained in order to protect confidentiality before, during, and after the trial	16
Declaration of interests	28	Financial and other competing interests for principal investigators for the overall trial and each study site	17
Access to data	29	Statement of who will have access to the final trial dataset, and disclosure of contractual agreements that limit such access for investigators	16
Ancillary and post-trial care	30	Provisions, if any, for ancillary and post-trial care, and for compensation to those who suffer harm from trial participation	16-17

For peer review only - http://bmjopen.bmj.com/site/about/guidelines.xhtml

Dissemination policy	31a	Plans for investigators and sponsor to communicate trial results to participants, healthcare professionals, the public, and other relevant groups (eg, via publication, reporting in results databases, or other data sharing arrangements), including any publication restrictions	16
	31b	Authorship eligibility guidelines and any intended use of professional writers	16
	31c	Plans, if any, for granting public access to the full protocol, participant-level dataset, and statistical code	N/A
**Appendices**			
Informed consent materials	32	Model consent form and other related documentation given to participants and authorised surrogates	Attached to submission, pg7 cited
Biological specimens	33	Plans for collection, laboratory evaluation, and storage of biological specimens for genetic or molecular analysis in the current trial and for future use in ancillary studies, if applicable	N/A

*It is strongly recommended that this checklist be read in conjunction with the SPIRIT 2013 Explanation & Elaboration for important clarification on the items. Amendments to the protocol should be tracked and dated. The SPIRIT checklist is copyrighted by the SPIRIT Group under the Creative Commons "Attribution-NonCommercial-NoDerivs 3.0 Unported" license.

For peer review only
